# Regulation of On-Tree Vitamin E Biosynthesis in Olive Fruit during Successive Growing Years: The Impact of Fruit Development and Environmental Cues

**DOI:** 10.3389/fpls.2016.01656

**Published:** 2016-11-16

**Authors:** Egli C. Georgiadou, Vlasios Goulas, Thessaloniki Ntourou, George A. Manganaris, Panagiotis Kalaitzis, Vasileios Fotopoulos

**Affiliations:** ^1^Department of Agricultural Sciences, Biotechnology and Food Science, Cyprus University of TechnologyLemesos, Cyprus; ^2^Department of Horticultural Genetics and Biotechnology, Mediterranean Agronomic Institute of ChaniaChania, Greece

**Keywords:** antioxidant capacity, relative transcription levels, tocopherols, tocotrienols, vitamin E, VTE5, Western blot

## Abstract

The term vitamin E refers to a group of eight lipophilic compounds known as tocochromanols. The tocochromanols are divided into two groups, that is, tocopherols and tocotrienols, with four forms each, namely α-, β-, γ-, and δ-. In order to explore the temporal biosynthesis of tocochromanols in olive (*Olea europaea* cv. ‘Koroneiki’) fruit during on-tree development and ripening over successive growing years, a combined array of analytical, molecular, bioinformatic, immunoblotting, and antioxidant techniques were employed. Fruits were harvested at eight successive developmental stages [10–30 weeks after flowering (WAF)], over three consecutive years. Intriguingly, climatic conditions affected relative transcription levels of vitamin E biosynthetic enzymes; a general suppression to induction pattern (excluding *VTE5*) was monitored moving from the 1^st^ to the 3^rd^ growing year, probably correlated to decreasing rainfall levels and higher temperature, particularly at the fruit ripening stage. A gradual diminution of VTE5 protein content was detected during the fruit development of each year, with a marked decrease occurring after 16 WAF. Alpha-tocopherol was the most abundant metabolite with an average percentage of 96.82 ± 0.23%, 91.13 ± 0.95%, and 88.53 ± 0.96% (during the 1^st^, 2^nd^, and 3^rd^ year, respectively) of total vitamin E content in 10–30 WAF. The concentrations of α-tocopherol revealed a generally declining pattern, both during the on-tree ripening of the olive fruit and across the 3 years, accompanied by a parallel decline of the total antioxidant capacity of the drupe. Contrarily, all other tocochromanols demonstrated an inverse pattern with lowest levels being recorded during the 1^st^ year. It is likely that, in a defense attempt against water deficit conditions and increased air temperature, transcription of genes involved in vitamin E biosynthesis (excluding *VTE5*) is up-regulated in olive fruit, probably leading to the blocking/deactivating of the pathway through a negative feedback regulatory mechanism.

## Introduction

Olive trees are common in the Mediterranean region and they are cultivated because of economic, health and nutritional reasons (Turktas et al., 2013). A plethora of polyphenolic compounds has been identified in olive fruits, such as oleuropein hydroxytyrosol, tyrosol, vanillic acid, and luteolin and their presence has been correlated with many claims for human health of olive fruits and its products (Soler-Rivas et al., 2000). Importantly, olive fruit contains significant amounts of tocochromanols that are known as potent lipophilic antioxidants and are essential dietary nutrients for mammals as vitamin E (Schneider, 2005). The latter consists of eight distinct forms organized in two chemical groups (α-, β-, γ-, δ-tocopherols and α-, β-, γ-, δ-tocotrienols, respectively). Tocopherols and tocotrienols are well-known health-promoting compounds, commonly found in several fruits and vegetables, including olive (Chun et al., 2006). Numerous studies have revealed several physiological responses to tocochromanols that may be relevant to the promotion of health and the prevention or treatment of some chronic diseases (Sen et al., 2007). Tocochromanols protect against cancer (Colombo, 2010), cardiovascular and neurological diseases (Aggarwal et al., 2010), and reduce the risk for Alzheimer’s disease (Wollen, 2010).

Alpha-tocopherol accounts for up to 68–89% of total tocopherols content in olive fruit depending on the cultivar and developmental stage (Muzzalupo et al., 2011). A large diversity in the concentration of tocochromanols in olive fruit has been described, highlighting the impact of genetic and environmental factors on tocochromanol composition (Hassapidou and Manoukas, 1993; Bruno et al., 2009; Muzzalupo et al., 2011; Bodoira et al., 2015).

Research on the biosynthetic pathway of vitamin E mainly focuses on plants such as *Solanum lycopersicum* L. (Quadrana et al., 2013), *Arabidopsis thaliana* (L.) Heynh (Li et al., 2010; Zhang et al., 2013, 2014), *Nicotiana tabacum* L. (Yabuta et al., 2013) and *Lactuca sativa* L. (Ren et al., 2011; Yabuta et al., 2013), while very little information exists regarding fruit tree crops, including olive (Georgiadou et al., 2015; Supplementary Figure S1). In our previous study (Georgiadou et al., 2015), the high resolution temporal gene expression profiles of tocochromanol biosynthetic genes were determined in parallel with the content of the eight vitamin E forms during 17 on-tree olive fruit developmental stages corresponding to an 8 month period starting in June and finishing in January. The current work aimed to determine the temporal transcript profiles of tocopherol and tocotrienol biosynthetic genes and the quantification of VTE5 protein content in parallel with tocochromanol composition and antioxidant capacity across consecutive years. The long term objective is the regulation of the biosynthetic pathway of tocochromanols in order to increase their content during olive fruit harvest for olive oil production. The samples were collected over eight on-tree developmental stages (10–30 WAF) of olive fruit (*Olea europaea* cv. Koroneiki) from three successive growing years, ultimately aiming to investigate any differences between consecutive growth years.

## Materials and Methods

### Fruit Material and Sampling Design

Olive fruits were harvested for three consecutive years from four olive trees (cv. ‘Koroneiki’) held at the experimental orchard of the Mediterranean Agronomic Institute of Chania (MAICh), Crete. Detailed meteorological data (temperature, rainfall, and relative humidity) throughout the cultivation years were recorded (Supplementary Figures S2 and S3; **Table 1**). The selected plants were productive olive trees (25–30 years old) of the cv. ‘Koroneiki’ with similar bearing habits and maturation. The average height of the four olive trees was 4.01 m and the average diameter of the crown was 4.87 m. The four olive trees had approximately the same size and the fruits they were bearing were approximately at the same stage. The olive trees were neither pruned nor fertilized or irrigated but they receive natural rainfall during the 3 year period of sampling. The trees were chosen and labeled, and the sampling of olive fruits was always performed between 9 and 10 a.m. All samples were systematically collected perimetrically from the crown, and at a height of 1.7 m. Approximately, 25 olive fruits per tree were collected and pooled (Roca and Mínguez-Mosquera, 2003). Subsequently, the mesocarp was separated from the seed, frozen in liquid nitrogen, grounded into fine powder using mortar and pestle, and stored at -80°C.

**Table 1:** Average air temperature (Tair) °C and total rainfall during 10–30 WAF for each of the three successive years in the experimental orchard.

Weeks after flowering (WAF)	Average air temperature (Tair) °C			Total rainfall (mm/cm^2^)		
	1^st^ year	2^nd^ year	3^rd^ year	1^st^ year	2^nd^ year	3^rd^ year
10	21.60	22.76	21.61	0.00	3.80	0.20
14	25.42	25.93	24.43	0.00	0.00	0.00
16	27.56	26.04	25.56	0.00	0.00	0.00
20	22.69	22.62	21.30	0.40	0.00	1.20
22	20.98	19.68	19.35	0.00	0.00	0.40
24	16.63	17.80	17.32	15.20	2.40	0.00
26	15.62	17.98	16.67	3.80	0.40	0.20
30	11.88	11.67	19.60	6.20	14.60	8.80

Initially, specimens were divided into eight seasonal phases from the middle of July to the beginning of December during three successive growth years (Supplementary Table S1). Full bloom was at the end of May for every successive year and corresponds to 0 WAF. Developmental stages (*S_1_*–*S_8_*) corresponded to 10–30 WAF (Supplementary Table S2) according to the phenological growth stages of olive trees as reported by the Biologische Bundesanstalt Bundessortenamt Chemische Industrie (BBCH) (Sanz-Cortes et al., 2002). The mesocarp developmental stage corresponds to 10–22 WAF, while the ripening stage of the olive fruit corresponds to 22–30 WAF (Conde et al., 2008; Alagna et al., 2009).

### RNA Extraction and rDNase Treatment

Total RNA was extracted from three independent bulked samples of 100 mg of olive tissue according to the protocol developed by Christou et al. (2014) and treated with RNase-free DNase (Cat. No. NU01a, HT Biotechnology LTD, England), in order to remove gDNA. Briefly, 0.5 μL (1 unit) of DNase I (RNase-free) was added to the extracts and ddH_2_O was added to a final volume of 50 μL. Samples were mixed and incubated at 37°C for 30 min. The enzyme was heat-inactivated at 75°C (5 min) and the volume was raised to 150 μL with the addition of ddH_2_O. Subsequently, 1/10 volume of 3 M CH_3_COONa (pH = 4.8) and 2.5 volumes of absolute ethanol were added, and the samples were briefly vortexed and incubated at -80°C overnight. RNA was precipitated by centrifugation at 16000 × *g* for 30 min at 4°C (Eppendorf Centrifuge 5415 R, Germany). The supernatant was discarded and the Eppendorf tubes were dried at 50°C for 2–3 min. RNA was dissolved in 20 μL ddH_2_O. The RNA integrity was examined spectrophotometrically (Nanodrop 1000 Spectrophotometer, Thermo Scientific, USA) and by gel electrophoresis before storing at -20°C.

### cDNA Synthesis and Quantitative RT-PCR Analysis

For the first-strand cDNA synthesis, 1 μg of total RNA was reversed transcribed using the PrimeScript^TM^ RT reagent Kit (Takara Bio, Japan) according to the manufacturer’s instructions. Quantitative RT-PCR (qRT-PCR) was performed using a Biorad IQ5 real-time PCR cycler (Bio-Rad, USA). In total, three biological replicates were performed for each seasonal phase for each year. The reaction mix contained 4 μL cDNA in reaction buffer (5-fold diluted first-strand cDNA), 0.5 μL of each primer (10 pmol/μL) and 5 μL 2X master mix (KAPA SYBRR FAST qPCR Kit, Kapa-Biosystems, USA). The total reaction volume was 10 μL. The initial denaturation stage was at 95°C for 5 min, followed by 40 cycles of amplification [95°C for 30 s, annealing temperature (Ta°C) for 30 s, and 72°C for 30 s] and a final elongation stage at 72°C for 5 min. Gene amplification cycle was followed by a melting curve run, carrying out 61 cycles with 0.5°C increment between 65 and 95°C. Tocochromanol biosynthetic primers were used (*VTE5, geranylgeranyl reductase, HPPD, VTE2, HGGT, VTE3, VTE1*, and *VTE4*) as reported by Georgiadou et al. (2015). The annealing temperature of the primers used ranged between 54 and 65°C as described by Georgiadou et al. (2015). The *UBQ2* gene was used as a housekeeping reference gene (Hernández et al., 2009).

### Phylogenetic Tree Analysis

The *O. europaea* VTE5 amino acid residues were queried for homology against known proteins in the NCBI database, employing the Blastp algorithm^1^. Forty nine proteins (Supplementary Table S3) that had similarity to the olive phytol kinase (and were also characterized as phytol kinases) were selected in order to construct a phylogenetic tree. An amino acid alignment was conducted using the MUSCLE algorithm and all positions that had gaps were removed using alignment curation. The Maximum Likelihood (ML) method and an approximate Likelihood-Ratio Test (aLRT) were selected for the construction of the dendrogram and for statistical support testing of branch lengths. All the above procedures were conducted using the “A la Carte” workflow as implemented in the http://phylogeny.lirmm.fr/phylo_cgi/index.cgi site, as reported by Dereeper et al. (2008). Visualization of the tree was conducted via the Treeview software (Page, 1996; Supplementary Figure S4).

### Raising Antibodies for VTE5, Protein Extraction and Immunoblotting of VTE5 Protein

In order to identify the VTE5 protein, *OeVTE5* EST sequence (Georgiadou et al., 2015) was queried using the ORF (open reading frame finder) analysis tool at the NCBI database^2^. Polyclonal antibodies (PAB) targeting the delivered protein were then raised (against the custom peptide RLLIHGLSLATDEGLVK) by Metabion GmbH (Germany).

Proteins were extracted from 0.5 g olive drupes using a Homex 6 Homogenizer (Bioreba, Switzerland) and 3 mL protein extraction buffer [0.05% w/v Tween 20, 2% w/v PVP, 5% v/v glycerol, 25 mM DTT, 1% v/v protease inhibitor cocktail dissolved in 1x PBS (pH 7.4, 8 g NaCl, 0.2 g KCl, 1.44 g Na_2_HPO_4_, 0.24 g KH_2_PO_4_)]. Protein concentration was determined using the Bradford assay (Bradford, 1976). Total protein extracts were mixed with 50 μL 4x SDS-sample buffer [which contained 2.0 mL (1 M) Tris-HCl pH 6.8, 0.8 g SDS, 4.0 mL (100% v/v) glycerol, 1.0 mL (0.5 M) EDTA pH 8, 8 mg bromophenol blue, up to 10 mL ddH_2_O and 14.7 M β-mercaptoethanol] for a total volume of 100 μL (ratio: 1:1). The PiNK Prestained Protein Ladder (NIPPON Genetics, EUROPE GmbH, MWP02, Europe) was used as a molecular weight standards. Each sample was heated at 95°C for 5 min and immediately cooled on ice. Proteins of 20 μL (12 μg per well) were separated by 12% separating gel and 5% stacking gel in SDS running buffer (144 g glycine, 30.2 g Tris base, 10 g SDS, diluted 10 times before use) at a constant voltage of 90 V for 3 h, using a Mini PROTEAN III apparatus (Bio-Rad, USA) before blotting on to PVDF membrane using a wet blotter at 35 V for 80 min. The transfer buffer contained 100 mL 10x transfer buffer [15.14 g Tris base, 72.06 g glycine, 20 mL (10% w/v) SDS], 200 mL methanol and 700 mL ddH_2_O.

The membrane was subsequently stained with 0.1% w/v Ponceau S in 5% v/v acetic acid to verify that proteins were transferred to the membrane, and then destained with wash buffer (0.05% v/v Tween 20 dissolved in 1x PBS). The membrane was blocked with blocking solution (2.5% w/v non-fat milk dissolved in wash buffer) for 1 h at room temperature and then washed three times with wash buffer (5 min each). The primary VTE5 antibody was diluted at a 1/500 ratio in the blocking solution and the membrane was incubated for 2 h at room temperature. It was then washed three times with wash buffer (5 min each). The membrane was incubated with the secondary antibody (anti-rabbit HRP conjugated from Sigma, Deutschland) (1/10000 dilution) in blocking solution for 1 h at room temperature, washed three times with wash buffer for 2 min each and enhanced chemiluminescence mixture (ECL) (Product 34077, Super Signal@WestPico, Thermo Scientific, USA) was added in it. The membrane was placed in an Infinite 1500/36M system (Vilber Lourmat, France), and a picture was taken under UV exposure.

### HPLC Analysis of Tocochromanols

A quantity of olive fruit (∼100 mg) was mixed with 1 mL acetonitrile-methanol-water (72/18/10, v/v/v) in 2-mL Eppendorf tube. The mixture was shaken in the dark for 15 min at 60°C using a Lab Companion SI-600R benchtop shaker following 5 min pre-incubation. Then, the mixture was centrifuged at 16000 × *g* for 5 min at 4°C (Eppendorf Centrifuge 5415 R, Germany) and the supernatant was collected and stored at -20°C until HPLC analysis (Gruszka and Kruk, 2007). The chromatographic separation was carried out on a Waters series HPLC system (Model “e2695”) equipped with vacuum degasser, quaternary pump, autosampler, thermostatted column compartment and multi l fluorescence detector. The data collection and analysis was performed by Empower software (Waters Corporation, Milford, Ireland). The extracts were loaded on a reverse phase XTerra RP18 (5 μm; 4.6 mm × 250 mm) column (Waters Corporation, Milford, Ireland). The flow rate was 0.8 ml min^-1^ and the injection volume was 20 μL. An isocratic elution was also performed using a mobile phase composed of acetonitrile/methanol/2-propanol (40/55/5, v/v/v). The elution of tocochromanols was recorded at an excitation wavelength of 292 nm and an emission wavelength of 335 nm (Tsochatzis et al., 2012). Their quantification was carried out using a six-level calibration curve for each of the studied tocochromanols.

### Determination of Antioxidant Capacity

In total, three techniques were used to determine the antioxidant capacity. The same extracts were used for the first two techniques: 1,1-Diphenyl-2-picrylhydrazyl (DPPH) and 2,2′-azino-bis(3-ethylbenzothiazoline-6-sulphonic acid) (ABTS). Antioxidants were extracted from the samples using the following procedure: two mL of ethanol/n-hexane (1/1, v/v) were added to 0.05 g of grounded plant tissue and vortexed. The mixtures were then placed in an ultrasonic bath for 5 min at 27°C and then shaken at 4°C for 48 h. Subsequently, samples were centrifuged for 10 min at 16000 × g at 4°C (Eppendorf Centrifuge 5415 R, Germany), and the supernatant was stored in sealed vials at -20°C for further analysis. For the third technique [ferric reducing ability of plasma (FRAP)], 0.05 g of ground plant tissue was weighed and 2 mL of n-hexane added. The preparation of the extracts is the same with DPPH and ABTS.

Three mL of freshly prepared FRAP solution (0.3 mol L^-1^ acetate buffer (pH 3.6) containing 10 mmol L^-1^ TPTZ and 40 mmol L^-1^ FeCl_3_^∗^10 H_2_O) were added to 100 μL of each *n*-hexane extract (0.05 g/2 mL) and shaken on a Polytron. Samples were incubated at 37°C for 4 min and centrifuged for 30 s at 16000 × *g* at 4°C (Eppendorf Centrifuge 5415 R, Germany) to separate the layers. Finally, the absorbance of the aqueous phase was measured at 595 nm, and the total antioxidant capacity was expressed as μmol α-tocopherol/g F.W. A calibration curve was constructed using freshly prepared α-tocopherol solutions (63–1000 μmol L^-1^) (Muller et al., 2011).

For the DPPH assay, 0.6 mL of extract [0.05 g/2 mL ethanol/*n*-hexane (1/1, v/v)] was mixed with 0.3 mL of 0.3 mmol L^-1^ ethanolic DPPH stock solution (stored in 4°C). The mixture was shaken, placed in the dark for 30 min and the absorbance of the solution was measured at 515 nm; the total antioxidant capacity was calculated as μmol α-tocopherol /g F.W. A calibration curve was constructed using freshly prepared α-tocopherol solutions (4.5–114 μmol L^-1^) (Muller et al., 2010).

ABTS radical cation (ABTS^.+^) was produced by reacting 7 mM ABTS stock solution with 2.45 mM potassium persulfate (final concentration) and allowing the mixture to stand overnight in the dark, at room temperature. After incubation, the solution was diluted with ethanol (lipophilic assay) to a 734 nm absorbance of 0.70 (± 0.02). For the photometric assay, 10 μL of antioxidant solution [0.05 g/2 mL ethanol/*n*-hexane (1/1, v/v)] were mixed for 45 s with 1 mL of diluted ABTS^⋅+^ solution and the optical density at 734 nm was determined after a 5 min incubation at 30°C. A calibration curve was constructed using freshly prepared α-tocopherol solutions (7.81–1000 μmol L^-1^). Results were expressed in μmol α-tocopherol/g F.W. (Jemai et al., 2009).

### Statistical Analysis

The relative transcript levels were calculated using the REST-XL software as reported by Pfaﬄ et al. (2002). UBQ2 gene was used as a housekeeping reference gene and the 10 WAF for each year was used for calibration.

Statistical analysis for the HPLC-Fluorescence detector analysis and the Antioxidant capacity assays were carried out using the software package SPSS v17.0 (SPSS, Inc., Chicago, IL, USA). The comparison of averages of each treatment was based on the analysis of variance according to Duncan’s multiple range Test at a 5% significance level (*P* ≤ 0.05). The results obtained through HPLC-Fluorescence detector analysis are presented as relative metabolite levels based on 10 WAF for each year.

## Results

### *In silico* Analysis of *VTE5* Gene Involved in the Biosynthetic Pathway of Vitamin E

BLAST analysis on NCBI and OLEA EST db databases resulted in the identification of a single cDNA for *VTE5* genes across several species. The deduced amino acid sequences of VTE5 revealed high similarities with homologs of other plants, suggesting that *VTE5* is conserved among plants (Supplementary Table S3). This was further illustrated through phylogenetic analysis of all examined components of the pathway, where *OeVTE5* grouped together with other VTE5 isoforms from other monocotyledonous and dicotyledonous plants (Supplementary Figure S4).

### Transcript Abundance of Vitamin E Biosynthetic Genes during Fruit Development and Ripening

**FIGURE 1:**
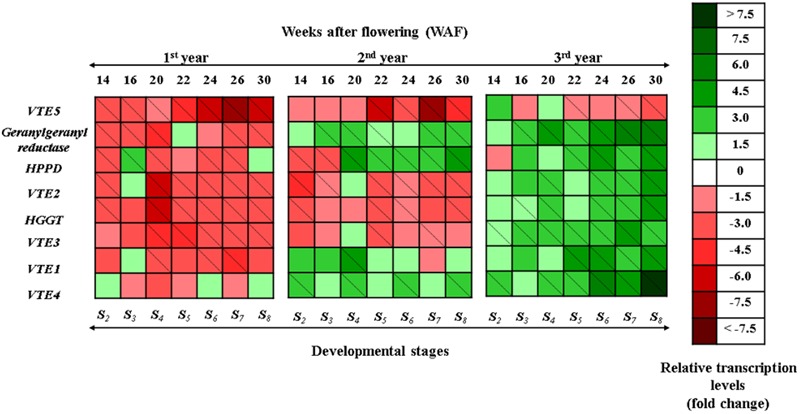
**Heat map showing temporal transcript expression pattern of the biosynthetic pathway of vitamin E in olive fruit (cv. “Koroneiki”) during 10–30 WAF for each of the three successive years in the experimental orchard.** Relative mRNA abundance was evaluated by real-time RT-PCR using three biological repeats. Up-regulation is indicated in green; down-regulation is indicated in red. A diagonal line in a box indicates a statistically significant value (*P* ≤ 0.05). A scale of color intensity is presented as a legend. Actual relative transcript values are shown in Supplementary Figures S5–S7. Values that differ from the seasonal phase 10 WAF for each year, used as reference.

During the 1^st^ year, a general down-regulation pattern was observed in tocopherol and tocotrienol biosynthetic genes in fruit flesh (**Figure 1**; Supplementary Figures S5–S7). The transcript levels of *VTE5* declined during mesocarp development (14–22 WAF) and ripening phase (22–30 WAF), with highest decrease being observed at the ripening phase in correlation with decreasing air temperature and increasing rainfall levels. Transcripts of *geranylgeranyl reductase* were down-regulated with the highest decrease being observed after 14–20 WAF (-2.49, -2.32, and -3.18 fold change). *HPPD* showed down-regulation in all examined WAF except 16 WAF, with highest decrease observed after 20 WAF. Transcript levels of *VTE2* and *HGGT* in the 1^st^ year presented a gradual decrease in all WAF. The highest reduction for both genes was observed at 20 WAF. Furthermore, *VTE3* mRNA levels were down-regulated with the highest decrease being observed at 20, 22, and 26 WAF in the 1^st^ year. *VTE1* and *VTE4* were down regulated throughout fruit development and ripening. Specifically, *VTE1* displayed the highest decrease at 20, 24, and 26 WAF. The pattern of *VTE4* transcripts can be considered unique among all the genes involved in the vitamin E pathway, due to the fluctuated pattern at the latter stages (**Figure 1**; Supplementary Figures S5–S7).

During the 2^nd^ year, *VTE5* mRNA levels exhibited similar patterns to the 1^st^ year; however, a transient up-regulation was found for specific genes (*geranylgeranyl reductase, HPPD, VTE1*, and *VTE4*) compared with the 1^st^ year (**Figure 1**; Supplementary Figures S5–S7). Notably, g*eranylgeranyl reductase* presented up-regulation, peaking at 20, 26, and 30 WAF in the 2^nd^ year. *HPPD* was down-regulated at 14 and 16 WAF and up-regulated from 20 WAF onwards. The highest transcription levels were observed at 20, 22, and 30 WAF, respectively. Transcript levels of *VTE2* and *HGGT* presented down-regulation, and the highest decrease for both genes was observed at 14 and 22 WAF. *VTE3* mRNA levels followed similar transcription pattern with the 1^st^ year and the highest decrease was recorded at ripening stage (22 and 26 WAF). *VTE1* and *VTE4* transcription in the 2^nd^ year showed up-regulation with the progress of on-tree developmental stages. Specifically, *VTE1* displayed the highest mRNA levels at 20 WAF, while *VTE4* transcription peaked at 20, 24, and 30 WAF.

In general, transcription of tocochromanol biosynthetic genes gradually increased throughout the developmental stages during the 3^rd^ year, except for *phytol kinase (VTE5*) gene (**Figure 1**; Supplementary Figures S5–S7). Transcript levels of *VTE5* exhibited comparable patterns to the 1^st^ and 2^nd^ year, where a significant suppression was observed after 22 WAF (ripening stage) and with highest suppression levels being observed at 30 WAF. Up-regulation was observed for *geranylgeranyl reductase* with highest levels found at 20, 24, 26, and 30 WAF. A similar transcription pattern was observed also for *HPPD* with peaks in transcription after 16 WAF, while only one stage (14 WAF) exhibited a suppressed transcription profile. Transcript levels of *VTE2* and *HGGT* were up-regulated throughout fruit development and ripening with the highest levels for both genes at 20, 24, and 30 WAF in the 3^rd^ year. *VTE3* transcription was constant from 14 WAF to 24 WAF but rapidly increased after 26 WAF until 30 WAF. In addition, *VTE1* had a higher mRNA accumulation at the end of mesocarp development and the ripening stage during the 3^rd^ year. Furthermore, this pattern of transcript abundance was comparable to the pattern of *geranylgeranyl reductase, VTE2, HGGT*, and *VTE4* genes. Finally, *VTE4* was up-regulated in all WAF with the highest levels detected after 24 WAF.

### Immunoblotting of VTE5 Protein Expression during Fruit Development and Ripening

**FIGURE 2:**
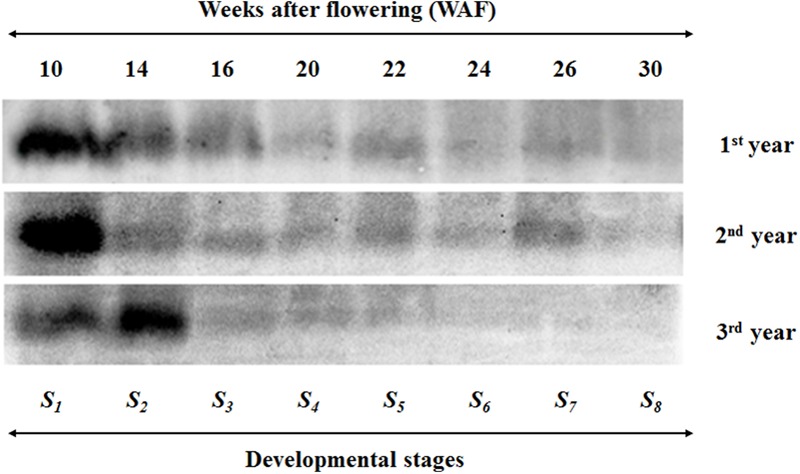
**Western blot analysis of olive fruit tissue indicating VTE5 protein content in the biosynthetic pathway of vitamin E in olive fruit (cv. ‘Koroneiki’) during 10–30 WAF for each of the three successive years in the experimental orchard.** The size of the VTE5 protein was approximately 30 kDa.

VTE5 protein content was determined for eight developmental stages over three successive growing years (**Figure 2**). The largest amounts of VTE5 protein were detected in the early developmental stages; 10, 14, and 16 WAF for the 1^st^ year and 10 and 14 WAF for the 2^nd^ and 3^rd^ year. For all years, the VTE5 protein content decreased constantly during mesocarp development (10–22 WAF) with a small increase at the breaker stage (22 WAF) for the 1^st^ and 2^nd^ year. The decrease then continued during olive fruit ripening (22–30 WAF), with the exception of a noticeable increase on 26 WAF of the 2^nd^ year. Across years, the 3^rd^ year showed the overall lowest VTE5 protein content. In particular, lowest quantities of VTE5 protein were detected during the ripening stage of the 3^rd^ year, concomitant with highest air temperature and lowest rainfall levels recorded between the three growth years.

### Quantification of Tocopherols and Tocotrienols during Fruit Development and Ripening

The abundance of α, β, γ, and δ forms of tocochromanols was determined in eight successive developmental stages of fruit development and on-tree ripening in cv. “Koroneiki” in three successive years, in order to study their temporal variation. All forms of tocopherols, α-tocotrienol and (β+γ)-tocotrienol were detected. Alpha-tocopherol was the most abundant form of tocochromanols in all 3 years with concentrations ranging between 10.38 and 32.65 mg/100 g F.W. compared with all the other tocopherols and tocotrienols with concentrations ranging between 0.16 and 0.76 mg/100 g F.W. Year after year, a small overall decrease was observed in α-tocopherol concentration. On the other hand (β+γ)-tocopherol concentrations increased slightly during the 2^nd^ and 3^rd^ year. The same increasing trend was observed for δ-tocopherol, α-tocotrienol and (β+γ)-tocotrienol. The α-tocotrienol concentration was non-detectable during the 1^st^ year, whereas small amounts were detected during the 2^nd^ and 3^rd^ year (**Table 2**).

**Table 2:** Metabolic content (mg/100 g F.W.) of the biosynthetic pathway of vitamin E in olive fruit (cv. ‘Koroneiki’) during 10–30 WAF for each of the three successive years in the experimental orchard.

Years	Metabolites	Metabolic content (mg/100 g F.W.) from 10 to 30 WAF							
		10	14	16	20	22	24	26	30
1^st^ year	α-tocopherol	31.48 ± 2.64	27.84 ± 1.66	27.11 ± 1.16	25.28 ± 1.23	27.01 ± 1.04	18.80 ± 0.72	18.36 ± 1.26	20.18 ± 0.44
	(β+γ)-tocopherol	0.36 ± 0.01	0.29 ± 0.03	0.28 ± 0.01	0.36 ± 0.01	0.51 ± 0.03	0.37 ± 0.02	0.34 ± 0.02	0.31 ± 0.02
	δ-tocopherol	0.19 ± 0.02	0.17 ± 0.02	0.21 ± 0.01	0.21 ± 0.01	0.28 ± 0.01	0.24 ± 0.01	0.20 ± 0.01	0.16 ± 0.01
	α-tocotrienol	nd^∗^	nd^∗^	nd^∗^	nd^∗^	nd^∗^	nd^∗^	nd^∗^	nd^∗^
	(β+γ)-tocotrienol	0.26 ± 0.02	0.25 ± 0.01	0.23 ± 0.03	0.20 ± 0.01	0.21 ± 0.02	0.20 ± 0.01	0.23 ± 0.03	0.18 ± 0.01
2^nd^ year	α-tocopherol	32.65 ± 3.65	24.28 ± 0.49	24.75 ± 3.53	22.75 ± 1.18	19.62 ± 1.26	15.87 ± 2.05	13.61 ± 0.87	14.03 ± 1.43
	(β+γ)-tocopherol	0.58 ± 0.07	0.55 ± 0.01	0.56 ± 0.04	0.59 ± 0.02	0.62 ± 0.03	0.59 ± 0.05	0.57 ± 0.04	0.60 ± 0.05
	δ-tocopherol	0.49 ± 0.06	0.47 ± 0.01	0.50 ± 0.03	0.50 ± 0.01	0.54 ± 0.03	0.54 ± 0.05	0.51 ± 0.03	0.56 ± 0.04
	α-tocotrienol	0.18 ± 0.02	0.18 ± 0.01	0.19 ± 0.01	0.19 ± 0.01	0.20 ± 0.01	0.20 ± 0.02	0.19 ± 0.01	0.21 ± 0.02
	(β+γ)-tocotrienol	0.59 ± 0.08	0.54 ± 0.01	0.57 ± 0.04	0.58 ± 0.02	0.61 ± 0.03	0.61 ± 0.05	0.57 ± 0.04	0.65 ± 0.05
3^rd^ year	α-tocopherol	24.90 ± 2.27	22.73 ± 0.44	14.73 ± 1.35	19.11 ± 0.13	10.38 ± 1.04	14.31 ± 0.39	13.19 ± 0.82	11.47 ± 0.50
	(β+γ)-tocopherol	0.57 ± 0.04	0.55 ± 0.01	0.63 ± 0.05	0.73 ± 0.01	0.52 ± 0.03	0.60 ± 0.02	0.61 ± 0.04	0.57 ± 0.01
	δ-tocopherol	0.53 ± 0.04	0.52 ± 0.01	0.59 ± 0.05	0.67 ± 0.01	0.48 ± 0.03	0.54 ± 0.02	0.56 ± 0.03	0.51 ± 0.01
	α-tocotrienol	0.20 ± 0.01	0.20 ± 0.01	0.22 ± 0.02	0.25 ± 0.01	0.18 ± 0.01	0.20 ± 0.01	0.21 ± 0.01	0.19 ± 0.01
	(β+γ)-tocotrienol	0.61 ± 0.04	0.62 ± 0.01	0.67 ± 0.05	0.76 ± 0.01	0.55 ± 0.04	0.60 ± 0.02	0.63 ± 0.04	0.57 ± 0.02

*Data are the means of three replications ± SE. ^∗^nd, non-detectable.*

**FIGURE 3:**
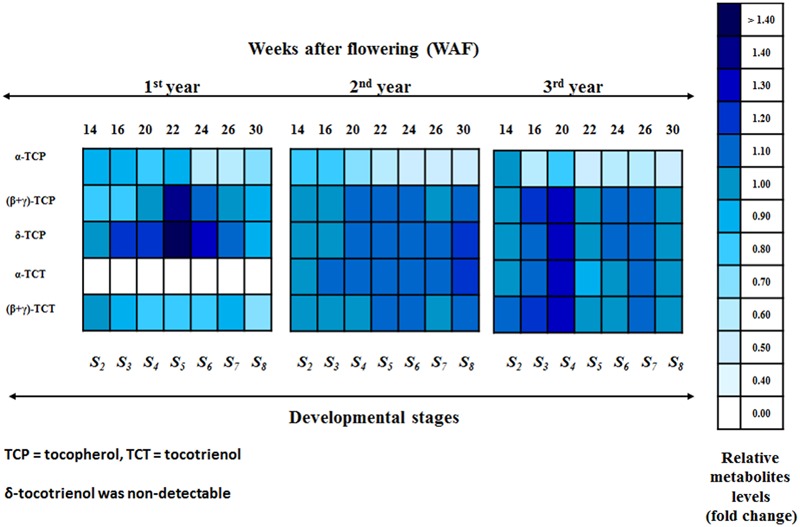
**Heat map of the relative metabolite levels of the biosynthetic pathway of vitamin E in olive fruit (cv. “Koroneiki”) during 10–30 WAF.** Relative metabolite levels were evaluated by HPLC using three biological repeats. A scale of color intensity is presented as a legend. Actual relative metabolite levels, obtained from three independent replicates, are shown in Supplementary Figures S8 and S9. Alpha-tocotrienol during the 1^st^ year and δ-tocotrienol were non-detectable. Relative metabolite levels based on 10 WAF for each year.

Regarding the 1^st^ year, all forms of tocopherols and (β+γ)-tocotrienol were detected (**Figure 3**; Supplementary Figures S8 and S9). The relative metabolite levels (compared with 10 WAF) of α-tocopherol showed a small decrease (0.80–0.88 fold change) within the period 14–22 WAF. After 22 WAF (early stages of fruit ripening), α-tocopherol levels decreased further up to 30 WAF (0.58–0.64 fold change). An interesting pattern was observed with the metabolite content of (β+γ)-tocopherol. At the early stages of mesocarp development (14 and 16 WAF), there was a decrease of metabolite levels followed by a return to the original concentration at 20 WAF, while further decreases were recorded during the late fruit ripening period (26 and 30 WAFs). On the other hand, the metabolite content of δ-tocopherol showed an increase in all WAFs, with the exception of 14 and 30 WAF. Similar to (β+γ)-tocopherol, the largest increase for δ-tocopherol was observed at 22 WAF (1.48- fold change). Regarding the tocotrienol content (**Figure 3**; Supplementary Figures S8 and S9), only (β+γ)-tocotrienol was detected, displaying declining metabolite levels for all WAFs, with the largest decline observed at 30 WAF (0.70- fold change).

For the 2^nd^ year, all forms of tocopherols and (β+γ)-tocotrienol were detected (**Figure 3**; Supplementary Figures S8 and S9). The relative metabolite levels (based on 10 WAF) of α-tocopherol showed slight decrease (0.60–0.76 fold change) during mesocarp development (14–22 WAF) and an even further decrease up to 30 WAF (0.42–0.49 fold change). On the other hand, the metabolite content of (β+γ)-tocopherol showed very little change across all WAFs, with minor increase during 20–24 and 30 WAF and minor decrease during 14, 16, and 26 WAF. A decrease in δ-tocopherol levels was observed at the early stages of mesocarp development (14 WAF), followed by a return to the original concentration at 16 WAF. Further increase was then observed after 20 WAF up to 30 WAF. The metabolite level trend for (β+γ)-tocotrienol was very similar to that of (β+γ)-tocopherol. There were very little changes across all WAFs with minor increases during 22, 24, and 30 WAF and decreases for the rest. The relative metabolite levels of α-tocotrienol showed a small but steady increase throughout the year (**Figure 3**; Supplementary Figures S8 and S9).

During the 3^rd^ year, all forms of tocopherols and (β+γ)-tocotrienol were again detected (**Figure 3**; Supplementary Figures S8 and S9). The relative metabolite levels (compared with 10 WAF) of α-tocopherol showed an overall high decline during all WAFs (0.42–0.91 fold change) with the lowest levels being recorded at 22 WAF. A decrease in (β+γ)-tocopherol levels was recorded at 14, 22 and 30 WAF, followed by an increase at 16–20 and 24–26 WAF. In the case of δ-tocopherol, a general increase was observed in all WAFs except 22 WAF where the levels were lower. The highest increase was observed at 20 WAF (1.25- fold change). A similar pattern was observed for (β+γ)-tocotrienol, with a general increase during all WAFs with the exception of 22 and 30 WAF. The highest increase was also observed at 20 WAF (1.25- fold change). On the other hand, the relative metabolite levels of α-tocotrienol revealed an interesting pattern in concentrations, which included an increase during 14–20 WAF (up to 1.23- fold change) followed by a sudden decline at 22 WAF and yet another small increase during 24–30 WAF (**Figure 3**; Supplementary Figures S8 and S9).

### Fluctuation of the Antioxidant Capacity among Different Developmental Stages and Years

In order to determine the antioxidant capacity of olive extracts, three different assays (FRAP, DPPH, and ABTS) were employed (Supplementary Figure S10). All assays indicated that antioxidant capacity was descending with the progress of on-tree developmental (10–22 WAF) and ripening (22–30 WAF) stages of olive fruits for all three growing years.

The FRAP assay showed a similar trend across all WAFs apart from a transient increase at 10 WAF during the 1^st^ year. On the contrary, the DPPH assay revealed an increase in antioxidant capacity at 10 and 14 WAF at all three growth years. The decrease from 10 WAF to 14 WAF in the 2^nd^ year was initially sharp, becoming gradual thereafter. Finally, an increase of antioxidant capacity at 10 and 14 WAF was detected in the 3 years with the ABTS assay, while an ongoing decrease in antioxidant capacity was detected after 16 WAF.

## Discussion

To explore the temporal biosynthesis of tocochromanols in olive fruit during on-tree development and ripening over a 3-year period, a combined array of analytical, molecular, immunoblotting, and antioxidant techniques was employed. For comparative purposes, part of the results from the 1^st^ year are included in a recent study (Georgiadou et al., 2015) that provided a high resolution temporal transcription profile of tocochromanol biosynthetic genes over 17 developmental stages. These developmental stages in olive fruit consist of a period of 8 months, starting in June and ending in January. This study focuses on the temporal transcription profiles of vitamin E genes and the quantification of VTE5 protein in parallel with tocochromanol composition and antioxidant capacity over three consecutive years and, hence, eight developmental stages (10–30 WAF) from each year were used.

A general down-regulation pattern was observed in tocochromanol biosynthetic genes (*VTE5, geranylgeranyl reductase, HPPD, VTE2, HGGT, VTE3, VTE1*, and *VTE4*) in fruit flesh during the 1^st^ year, followed by significant up-regulation of specific genes (*geranylgeranyl reductase, HPPD, VTE1*, and *VTE4*) during the 2^nd^ and 3^rd^ year an overall up-regulation of tocochromanol biosynthetic genes (with the exception of *VTE5*) (**Figure 1**; Supplementary Figures S5–S7). The transcription of *VTE5* was down-regulated during mesocarp development (14–22 WAF) and ripening phase (22–30 WAF), with the highest decrease observed at the ripening phase over a 3-year period. Interestingly, a significant drop in transcription was recorded at breaker stage (22 WAF). This drop was lower than the calibrator (10 WAF) and sustained up to 30 WAF. A similar transition in concentration was observed for tocopherols and tocotrienols (and especially in α-tocopherol) with significantly higher amounts until 22 WAF (for the 1^st^ and 2^nd^ year) and 20 WAF (for the 3^rd^ year) and much lower thereafter, indicating a tight correlation with the transcription profile of *VTE5*. Moreover, oil production in the olive fruit reaches its maximum at the end of the mesocarp development (Conde et al., 2008; Alagna et al., 2009; Bodoira et al., 2015). Similar results can be found in a recent study on tomato fruit (Quadrana et al., 2013) and on olive fruit (Georgiadou et al., 2015), where a reduction of *VTE5* transcript is connected with ripening. The *VTE5* reduction directly limits phytol diphosphate [Phytyl-PP (PDP)] input supply toward vitamin E biosynthesis (Quadrana et al., 2013) and relates with a reduction of tocochromanol content during the development and maturity of the olive fruit. Therefore, *VTE5* appears to be a key player in the biosynthesis of vitamin E in olive fruit and is thus proposed as a marker gene in relevant studies.

Similar results with transcription of *VTE5* were observed in VTE5 protein content (**Figure 2**). The largest amounts of VTE5 protein were detected in the early developmental stages; 10, 14 and 16 WAF for the 1^st^ year and 10 and 14 WAF for the 2^nd^ and 3^rd^ year. For all years, the VTE5 protein content decreased constantly during mesocarp development (10–22 WAF) with a small increase at the breaker stage (22 WAF) for the 1^st^ and 2^nd^ year. The decrease then continued during olive fruit ripening (22–30 WAF), with the exception of a noticeable increase on 26 WAF of the 2^nd^ year. Across years, the 3^rd^ year showed the overall lowest VTE5 protein content. Transcript levels of *VTE5* exhibited a similar behavior in the first and 2^nd^ years, where significant down-regulation were observed in all WAFs with a significant drop in transcription after 22 WAF (with sharpest declines being recorded at 26 WAF of the 2^nd^ year) and sustained up to 30 WAF (the results of VTE5 protein content also depicted decrease of the protein along with the development and maturity of the olive fruit). It is possible that VTE5 protein molecule could have longer half-life and shorter turnover rate compared with respective mRNAs, in line with universal molecule property trends previously demonstrated which show that proteins have in general higher mean half-life in comparison with corresponding mRNAs (Schwanhäusser et al., 2011). Furthermore, there is the possibility that additional VTE5 isoforms exist, either as alleles of the same gene or as gene products of additional VTE5 genes. In this case, the VTE5 protein content cannot be solely attributed to the VTE5 gene expression. However, the transcription levels of *VTE5* for 14 WAF were up-regulated in the 3^rd^ year in contrast with previous years (being in agreement with large amounts of VTE5 protein in 14 WAF). Vitamin E content decreases slightly until the end of mesocarp development and then decreases sharply during the ripening of the olive fruit (22–30 WAF). This pattern is consistent for all 3 years, providing evidence that VTE5 pays an important role. The chlorophyll content in fruit mesocarp also drops during this period (Alagna et al., 2009). Olive oil production, however, decreases after 22 WAF along with vitamin E content. At the beginning of the breaker stage (22 WAF) the processes of carbohydrate metabolism, fatty acid biosynthesis, and triacylglycerols (TAGs) are more evident and may affect oil and vitamin E content. Phytol can be transformed in phytyl-P, followed by phytyl-PP, and ending in producing chlorophyll, phylloquinone (vitamin K) and tocopherols. Furthermore, the active fatty acyl group can restrict the free phytol through the reaction of acyltransferase and produce fatty acid phyrol ester synthesis (Ischebeck et al., 2006). Related studies report that chlorophyll degradation leads to the majority of phytyl-PP for tocopherol biosynthesis in *Arabidopsis* seeds (Ischebeck et al., 2006; Valentin et al., 2006).

Alpha-tocopherol was the most abundant form of tocochromanols in all 3 years compared with all other tocopherols and tocotrienols, in accordance with previous studies (Hassapidou and Manoukas, 1993; Bruno et al., 2009; Muzzalupo et al., 2011; Bodoira et al., 2015). The concentrations of α-tocopherol revealed a generally decreasing pattern, both within and across years (**Table 2**). High amounts of tocochromanol content have been reported during the early stages of olive fruit (cv. ‘Arauco’) development, followed by a decreasing pattern with the progress of on-tree fruit development (Bodoira et al., 2015). Within each year, the highest concentrations of α-tocopherol were observed during the period 10–22 WAF (fruit development) with maximum content detected at 10 WAF (**Figure 3**; Supplementary Figures S8 and S9). Alpha-tocopherol content decreased sharply at the early stages of fruit ripening, after 22 WAF, and remained at such levels up to 30 WAF. It has been observed by a number of reports that olive oil production and phenolic fraction increase and reach their highest levels toward the end of the mesocarp developmental (22 WAF), connected with the initiation of color change (Conde et al., 2008; Alagna et al., 2009; Sakouhi et al., 2011; Bodoira et al., 2015). Furthermore, carbohydrate metabolism (glycolysis/glyconeogenesis, citrate cycle, fructose, manose, and galactose metabolism) is more prevalent during the mesocarp development.

Concentration of all tocochromanols except α-tocopherol was generally lower during the 1^st^ year in comparison with that recorded during the 2^nd^ and 3^rd^ year. However, given the overwhelming abundance of α-tocopherol (∼92.16 ± 0.71% of total tocochromanols over 3 years), the total concentration of tocochromanols was decreased year after year. These results were similar to Muzzalupo et al. (2011) who found that α-tocopherol accounts for up to 68–89 % of total tocopherol content in olive fruit depending on the cultivar and developmental stage. Results were also in agreement with Georgiadou et al., 2015 who showed that α-tocopherol accounts for 95.6–97.9% in cv. ‘Koroneiki’ of total tocochromanols content. The difference in comparison with findings by Bodoira et al. (2015) is that γ-tocopherol concentrations are only slightly lower than those of α-tocopherol at the early stages of (cv. ‘Arauco’) olive fruit. In a related work, it was found that α-tocopherol accounts for 90.9% of all other tocopherols in tomato fruit (Quadrana et al., 2013) and more than 80% of all other tocochromanols in carrot cultivars (Luby et al., 2014). Furthermore, examination of α- and γ-tocopherol content in commercial capers revealed that α-tocopherol was the predominant compound in all samples with an average content of 66.9% (Tlili et al., 2011). Importantly, it was also observed that in grape, a non-climacteric fruit like olive, tocopherol content declined gradually during its development (Horvath et al., 2006). An inverse pattern, however, is observed in climacteric fruits, such as mango (Singh et al., 2011) and tomato (Quadrana et al., 2013), thus potentially also implicating ethylene as a regulatory molecule of vitamin E biosynthesis. This hypothesis need to be further elucidated.

Several studies have examined the high antioxidant capacity of vitamin E with special reference on α-tocopherol (Ohkatsu et al., 2001; Karmowski et al., 2015). The particular chemical structure of α-tocopherol, which has a high degree of methylation in ortho-positions of the chromanol ring, intensifies its antioxidant capacity. Similarly, this study focuses and validates the high antioxidant capacity of α-tocopherol, especially during the early developmental stages. To this end, multiple assays have been used to determine antioxidant capacity, since a one-dimensional method is not adequate to investigate the antioxidant capacity (Goulas et al., 2012). In the current study, all three assays that were employed (FRAP, DPPH, and ABTS) showed similar results (Supplementary Figure S10). In addition to the high antioxidant capacities observed, a decreasing pattern was detected with the progress of fruit development in all years examined. This result could be potentially explained by previous studies that have correlated the decrease of antioxidant capacity during olive fruit maturation with the decomposition of oleuropein to elenolic acid glucoside or demethyloleuropein by endogenous esterases (Goulas et al., 2012). Nevertheless, total tocochromanol content of olive fruit (cv. ‘Koroneiki’) over 3 years presented moderate correlation (*R* = 0.750 and *R* = 0.636) with antioxidant capacity (using TEAC and DPPH, respectively). A weak correlation (*R* = 0.454) was monitored between FRAP values and total tocochromanol content. The observed correlation could be attributed to the fact that several phenolic compounds, namely hydroxytyrosol, tyrosol esters and *o*-diphenol, may contribute to the overall antioxidant capacity of olive fruit extract (Jemai et al., 2009).

Environmental conditions appear to possess an important role in olive fruit composition in tocochromanols. In particular, the 1^st^ year is characterized by sufficient water levels at ripening stage (total rainfall 25.20 mm/cm^2^ for 22–30 WAF), which gradually decreased during the 2^nd^ year (total rainfall 17.40 mm/cm^2^ for 22–30 WAF) reaching lowest (and potentially stressful) levels during the 3^rd^ year (total rainfall 9.40 mm/cm^2^ for 22–30 WAF) (**Table 1**; Supplementary Figure S2). Similarly, temperature was lowest during the 1^st^ year (average air temperature 16.28°C for 22–30 WAF), which slightly increased during the 2^nd^ year (average air temperature 16.78°C for 22–30 WAF) but showed about 2°C temperature increase in the ripening phase reaching highest levels during the 3^rd^ year (average air temperature 18.23°C for 22–30 WAF) (**Table 1**; Supplementary Figure S2). It is possible that, in a defense attempt against water deficit conditions and increased air temperature, transcription of genes involved in vitamin E biosynthesis (excluding *VTE5*) is up-regulated in olive fruit, probably leading to the blocking/deactivating of the pathway through a negative feedback regulatory mechanism (Antoniou et al., 2013) which results in decreasing tocochromanols, VTE5 content and antioxidant capacity as the olive fruit develops to maturity.

Past work on plant abiotic stress tolerance has shown decreases in net tocopherol levels resulting from increasing stress factors such as drought, especially on stress-sensitive plants such as *Salvia officinalis* (Munné-Bosch et al., 2001), while Pék et al. (2014) demonstrated increases in α-tocopherol content of rain fed cherry tomatoes. Contrarily, Loyola et al. (2012) demonstrated that α-tocopherol biosynthesis is part of the adaptation mechanisms to drought stress of *Solanum chilense*, a wild tomato genotype naturally growing in water deficit conditions. The observed correlation between reduced α-tocopherol content under increasingly stressful environmental conditions during the three growth years (lower rainfall levels, increased temperature) could however, propose another possible protection scenario in the form of increasing amounts of α-tocotrienol. Previous reports have established that a-tocochromanols have the highest antioxidant effectiveness among all tocochromanols due to their potency as hydrogen donors to lipid-free radicals (Kamal-Eldin and Appelqvist, 1996). While α-tocopherol and α-tocotrienol have equal antioxidant capacity *in vitro* when analyzed in hexane, it has been proposed that α-tocotrienol has a higher antioxidant potency than α-tocopherol, due to the combined effects of higher recycling efficiency, a more uniform distribution in the membrane bilayer, and a stronger disordering effect on membrane lipids (Kamal-Eldin and Appelqvist, 1996). It is therefore plausible that the plants ‘compensate’ for the lowering amounts of α-tocopherol by increasing α-tocotrienol content in olive fruit as environmental conditions become more stressful in an attempt to provide antioxidant protection, a role further supported by the tocotrienols’ almost exclusive localization in seeds and fruits (Falk and Munné-Bosch, 2010). However, it should be pointed out that overall findings suggest the more pronounced developmental regulation of tocopherol content in olive fruit rather than the regulation by environmental conditions. This regulation might be attributed partly at the transcriptional as well as post-transcriptional levels according to our gene expression and protein levels results. The higher expression levels compared with lower tocopherols content in the three consecutive years suggest the involvement of putative post-transcriptional regulation events.

Finally, it is worth noting that another possible explanation for the trends observed in the present study could be due to alternate bearing, a phenomenon observed in olive trees and which is sensitive to various environmental factors, although no production data are available to substantiate this. Olive trees have a high production year (“on year”) but a reduced one the following year (“off year”) (Lavee, 2007; Turktas et al., 2013). Empirical observations in the experimental orchard suggest that production was further decreased during the 3^rd^ year in comparison with the 1^st^ and 2^nd^ years, while fruit ripened earlier. The significant reduction of tocochromanols, VTE5 content and antioxidant capacity in olives could therefore be partly explained by the quicker ripening observed in the 3^rd^ year, which causes earlier chlorophyll degradation and more evident carbohydrate metabolism, fatty acid and triacylglycerols (TAGs) biosynthesis. In addition, stressful environmental conditions could also account for the lower olive production observed during the 3^rd^ year (Lavee, 2007; Turktas et al., 2013).

## Conclusion

This study describes the temporal characterization of vitamin E biosynthesis in olive fruit during on-tree development and ripening over a 3-year growth period. The key observed motifs from 1^st^ to 3^rd^ year were that gene transcription levels shifted from overall suppression to induction levels (excluding *VTE5*), while VTE5 protein content, tocochromanol content and antioxidant capacity decreased both across developmental stages and across the three successive years. In addition to the effect of developmental cues, the observed trends correlate with varying environmental conditions with gradually increasing air temperature and decreasing rainfall levels, particularly during the fruit ripening phase, over the 3 year period. It is therefore proposed that, in an attempt to respond to stressful conditions, the plant induces the transcription of most genes involved in vitamin E biosynthesis, likely leading to blocking of the pathway via a negative feedback regulatory mechanism, which ultimately leads to reduced tocochromanol content and overall antioxidant capacity. The present findings therefore highlight the important regulatory role of environmental conditions during vitamin E biosynthesis. Further research is required to address these issues such as exposure of olive fruits to heat stress and drought stress and investigation of how these conditions alter the tocopherols biosynthetic pathway.

## Author Contributions

Conceived and designed the experiments: GM, PK, and VF. Performed the experiments: EG, VG, and TN. Analyzed the data: EG, VG, and VF. Wrote the paper: EG, GM, PK, and VF. All authors have read and approved the manuscript.

## Conflict of Interest Statement

The authors declare that the research was conducted in the absence of any commercial or financial relationships that could be construed as a potential conflict of interest.

## Footnotes

1 http://blast.ncbi.nlm.nih.gov/Blast.cgi

2 http://www.ncbi.nlm.nih.gov/gorf/gorf.html

## Abbreviations

DMGGBQT: 2,3-dimethyl-6-geranylgeranyl-1,4-benzoquinol
DMPBQT: 2,3-dimethyl-6-phytyl-1,4-benzoquinol
GGPP or GGDPT: geranylgeranyl pyrophosphate or geranylgeranyl diphosphate
HGAT: homogentisic acid
HGGTT: homogentisate geranylgeranyl transferase
HPPT: p- or 4-hydroxyphenylpyruvic acid
HPPDT: p- or 4-hydroxyphenylpyruvate dioxygenase
HPT or VTE2T: homogentisate phytyl transferase or vitamin E2
MGGBQT: 2-methyl-6-geranylgeranylbenzoquinol
MPBQT: 2-methyl-6-phytylbenzoquinol
MPBQ MT or VTE3T: 2-methyl-6-phytyl-1,4-benzoquinol methyl transferase or vitamin E3
phytyl-P or PMPT: phytyl phosphate
phytyl-PP or PDPT: phytyl diphosphate
TC or VTE1T: tocopherol cyclase or vitamin E1
TPTZT: 2,4,6-tripyridyl-s-triazine
UBQ2T: polyubiquitin2
VTE5T: phytol kinase or vitamin E5
WAFT: weeks after flowering
γ-TMT or VTE4T: γ-tocopherol methyl transferase or vitamin E4
