# Supplementary material for: Regulation of On-Tree Vitamin E Biosynthesis in Olive Fruit during Successive Growing Years: The Impact of Fruit Development and Environmental Cues
Source: Front Plant Sci. 2016 Nov 16;7:1656. doi: 10.3389/fpls.2016.01656 (PMC5111394; doi:10.3389/fpls.2016.01656)
Supplement: Supplementary file 1 [file Data_Sheet_1.DOC]

**Supplementary Material**

**Regulation of On-Tree Vitamin E Biosynthesis in Olive Fruit during Successive Growing Years: The Impact of Fruit Development and Environmental Cues**

Egli C. Georgiadou, Vlasios Goulas, Thessaloniki Ntourou, George A. Manganaris, Panagiotis Kalaitzis, Vasileios Fotopoulos

**Supplementary Table S1.** Harvesting data on olive fruit of 'Koroneiki' cultivar during the eight developmental stages (10-30 WAF) of the olive fruits for the three successive years are indicated.

| **Developmental stages** | **1^st^ year** | **2^nd^ year** | **3^rd^ year** | **Weeks After Flowering**  **(WAF)** |
| --- | --- | --- | --- | --- |
| ***S_1_*** | 12 July | 11 July | 17 July | **10** |
| ***S_2_*** | 9 August | 9 August | 1 August | **14** |
| ***S_3_*** | 23 August | 22 August | 18 August | **16** |
| ***S_4_*** | 20 September | 19 September | 18 September | **20** |
| ***S_5_*** | 4 October | 3 October | 3 October | **22** |
| ***S_6_*** | 18 October | 17 October | 15 October | **24** |
| ***S_7_*** | 1 November | 31 October | 2 November | **26** |
| ***S_8_*** | 29 November | 29 November | 3 December | **30** |

**Supplementary Table S2.** Description of 10-30 WAF of olive trees along with the phenological growth stage according to the BBCH (Biologische Bundesanstalt, Bundessortenamt, Chemische Industrie) scale. The mesocarp development phase corresponds to 10-22 WAF while the ripening phase of the olive fruit to 22-30 WAF.

| **Develop-mental stages** | **Phenological growth stage** | | **Weeks after flowering (WAF)** | | | | | | | |
| --- | --- | --- | --- | --- | --- | --- | --- | --- | --- | --- |
|  |  |  | **10** | **14** | **16** | **20** | **22** | **24** | **26** | **30** |
| ***S_1_ – S_3_*** | **75** | **Fruit about 50% of final size, stone becomes lignified** |  |  |  |  |  |  |  |  |
| ***S_4_*** | **79** | **Fruit about 90% of final size, fruit suitable for picking green** |  |  |  |  |  |  |  |  |
| ***S_5_* – *S_6_*** | **81** | **Beginning of fruit colouring** |  |  |  |  |  |  |  |  |
| ***S_7_*** | **85** | **Increasing specific fruit colouring** |  |  |  |  |  |  |  |  |
| ***S_8_*** | **89** | **Harvest maturity** |  |  |  |  |  |  |  |  |
|  | | | **🡨 mesocarp development 🡪** | | | | | **🡨 ripening 🡪** | | |

**Supplementary Table S3.** Protein homology of olive *VTE5* involved in the biosynthetic pathway of vitamin E.

| **Gene** | **Accession Number** | **Plant Species** | **Percentage**  **Identity** | **Percentage**  **Similarity** | **E-value** |
| --- | --- | --- | --- | --- | --- |
| *VTE5* | [XP_002269950.](http://www.ncbi.nlm.nih.gov/protein/225448861?report=genbank&log$=prottop&blast_rank=2&RID=X36T70NY014)1 | *Vitis vinifera* | 83 % | 83 % | 1e-11 |
|  | [XP_010665628.1](http://www.ncbi.nlm.nih.gov/protein/731370145?report=genbank&log$=prottop&blast_rank=4&RID=X36T70NY014) | *Beta vulgaris subsp. vulgaris* | 71 % | 97 % | 3e-11 |
|  | [XP_008218941.1](http://www.ncbi.nlm.nih.gov/protein/645224089?report=genbank&log$=prottop&blast_rank=5&RID=X36T70NY014) | *Prunus mume* | 81 % | 83 % | 3e-11 |
|  | [XP_008375637.1](http://www.ncbi.nlm.nih.gov/protein/657967897?report=genbank&log$=prottop&blast_rank=8&RID=X36T70NY014) | *Malus domestica* | 81 % | 83 % | 1e-10 |
|  | XP_011024295.1 | *Populus euphratica* | 75 % | 88 % | 2e-10 |
|  | XP_004159238.1 | *Cucumis sativus* | 78 % | 83 % | 5e-10 |
|  | [XP_009591844.1](http://www.ncbi.nlm.nih.gov/protein/697166062?report=genbank&log$=prottop&blast_rank=15&RID=X36T70NY014) | *Nicotiana tomentosiformis* | 81 % | 83 % | 6e-10 |
|  | [EEF29465](http://www.ncbi.nlm.nih.gov/protein/255584378?report=genbank&log$=prottop&blast_rank=17&RID=X36T70NY014).1 | *Ricinus communis* | 75 % | 83 % | 1e-09 |
|  | KHN00934.1 | *Glycine soja* | 72 % | 83 % | 2e-09 |
|  | [XP_011094481.1](http://www.ncbi.nlm.nih.gov/protein/747093358?report=genbank&log$=prottop&blast_rank=19&RID=X36T70NY014) | *Sesamum indicum* | 75 % | 83 % | 2e-09 |
|  | [KDP45055](http://www.ncbi.nlm.nih.gov/protein/802547436?report=genbank&log$=prottop&blast_rank=21&RID=X36T70NY014).1 | *Jatropha curcas* | 78 % | 83 % | 3e-09 |
|  | [XP_006588440.1](http://www.ncbi.nlm.nih.gov/protein/571480811?report=genbank&log$=prottop&blast_rank=22&RID=X36T70NY014) | *Glycine max* | 72 % | 83 % | 3e-09 |
|  | ERP57294.1 | *Populus trichocarpa* | 71 % | 81 % | 3e-09 |
|  | [XP_006364441.1](http://www.ncbi.nlm.nih.gov/protein/565397736?report=genbank&log$=prottop&blast_rank=24&RID=X36T70NY014) | *Solanum tuberosum* | 81 % | 83 % | 4e-09 |
|  | XP_011094482.1 | *Sesamum indicum* | 75 % | 83 % | 4e-09 |
|  | [XP_009783010.1](http://www.ncbi.nlm.nih.gov/protein/698466854?report=genbank&log$=prottop&blast_rank=30&RID=X36T70NY014) | *Nicotiana sylvestris* | 78 % | 83 % | 7e-09 |
|  | [ESW16295](http://www.ncbi.nlm.nih.gov/protein/593687283?report=genbank&log$=prottop&blast_rank=32&RID=X36T70NY014).1 | *Phaseolus vulgaris* | 72 % | 83 % | 8e-09 |
|  | XP_008456566.1 | *Cucumis melo* | 75 % | 83 % | 8e-09 |
|  | [KDO55302](http://www.ncbi.nlm.nih.gov/protein/567888122?report=genbank&log$=prottop&blast_rank=34&RID=X36T70NY014).1 | *Citrus sinensis* | 73 % | 86 % | 1e-08 |
|  | [XP_004234914.1](http://www.ncbi.nlm.nih.gov/protein/460378306?report=genbank&log$=prottop&blast_rank=37&RID=X36T70NY014) | *Solanum lycopersicum* | 81 % | 83 % | 2e-08 |
|  | [XP_010067215.1](http://www.ncbi.nlm.nih.gov/protein/702423130?report=genbank&log$=prottop&blast_rank=42&RID=X36T70NY014) | *Eucalyptus grandis* | 75 % | 83 % | 4e-08 |
|  | [XP_006653001.1](http://www.ncbi.nlm.nih.gov/protein/573941213?report=genbank&log$=prottop&blast_rank=40&RID=X36T70NY014) | *Oryza brachyantha* | 66 % | 88 % | 4e-08 |
|  | ACP43458.1 | *Lactuca sativa* | 67 % | 83 % | 5e-07 |
|  | [ABA42672.1](http://www.ncbi.nlm.nih.gov/protein/76443929?report=genbank&log$=prottop&blast_rank=60&RID=X36T70NY014) | *Zea mays* | 66 % | 88 % | 9e-07 |
|  | EOY18924.1 | *Theobroma cacao* | 69 % | 83 % | 8e-07 |
|  | EOY18925.1 | *Theobroma cacao* | 69 % | 83 % | 7e-07 |
|  | XP_010552940.1 | *Tarenaya hassleriana* | 64 % | 83 % | 7e-07 |
|  | ABA42672.1 | *Zea mays* | 66 % | 88 % | 8e-07 |
|  | [XP_004496158.1](http://www.ncbi.nlm.nih.gov/protein/502118218?report=genbank&log$=prottop&blast_rank=62&RID=X36T70NY014) | *Cicer arietinum* | 64 % | 83 % | 1e-06 |
|  | [KEH43364.1](http://www.ncbi.nlm.nih.gov/protein/657404741?report=genbank&log$=prottop&blast_rank=63&RID=X36T70NY014) | *Medicago truncatula* | 67 % | 83 % | 3e-06 |
|  | [XP_009396489.1](http://www.ncbi.nlm.nih.gov/protein/694996874?report=genbank&log$=prottop&blast_rank=65&RID=X36T70NY014) | *Musa acuminata subsp. malaccensis* | 67 % | 83 % | 3e-06 |
|  | XP_004960241.1 | *Setaria italica* | 63 % | 88 % | 3e-06 |
|  | [XP_008795888.1](http://www.ncbi.nlm.nih.gov/protein/672143993?report=genbank&log$=prottop&blast_rank=69&RID=X36T70NY014) | *Phoenix dactylifera* | 58 % | 83 % | 3e-06 |
|  | XP_009396497.1 | *Musa acuminata subsp. malaccensis* | 67 % | 83 % | 3e-06 |
|  | [XP_008795886.1](http://www.ncbi.nlm.nih.gov/protein/672143993?report=genbank&log$=prottop&blast_rank=69&RID=X36T70NY014) | *Phoenix dactylifera* | 58 % | 83 % | 1e-05 |
|  | DAA35443.1 | *Zea mays* | 64 % | 83 % | 1e-05 |
|  | [CDX70220.1](http://www.ncbi.nlm.nih.gov/protein/685379801?report=genbank&log$=prottop&blast_rank=76&RID=X36T70NY014) | *Brassica napus* | 56 % | 83 % | 5e-05 |
|  | ABA42674.1 | *Triticum aestivum* | 58 % | 88 % | 9e-05 |
|  | AED90752.1 | *Arabidopsis thaliana* | 58 % | 88 % | 6e-04 |
|  | [XP_010452347.1](http://www.ncbi.nlm.nih.gov/protein/727560253?report=genbank&log$=prottop&blast_rank=88&RID=X36T70NY014) | *Camelina sativa* | 56 % | 83 % | 0.001 |
|  | AFB74217.1 | *Brassica napus* | 53 % | 83 % | 0.001 |
|  | [XP_003580789.1](http://www.ncbi.nlm.nih.gov/protein/357166664?report=genbank&log$=prottop&blast_rank=90&RID=X36T70NY014) | *Brachypodium distachyon* | 53 % | 88 % | 0.002 |
|  | XP_010423571.1 | *Camelina sativa* | 53 % | 88 % | 0.002 |
|  | XP_010490961.1 | *Camelina sativa* | 53 % | 83 % | 0.002 |
|  | KHN02090.1 | *Glycine soja* | 50 % | 88 % | 0.054 |
|  | ABA42677.1 | *Glycine max* | 50 % | 88 % | 0.056 |
|  | XP_004500293.1 | *Cicer arietinum* | 45 % | 88 % | 0.42 |
|  | XP_004500292.1 | *Cicer arietinum* | 45 % | 88 % | 0.42 |
|  | [XP_004307151.1](http://www.ncbi.nlm.nih.gov/protein/470142948?report=genbank&log$=prottop&blast_rank=102&RID=X36T70NY014) | *Fragaria vesca subsp. vesca* | 72 % | 83 % | 0.66 |
|  | XP_010264775.1 | *Nelumbo nucifera* | 42 % | 88 % | 8.1 |

*
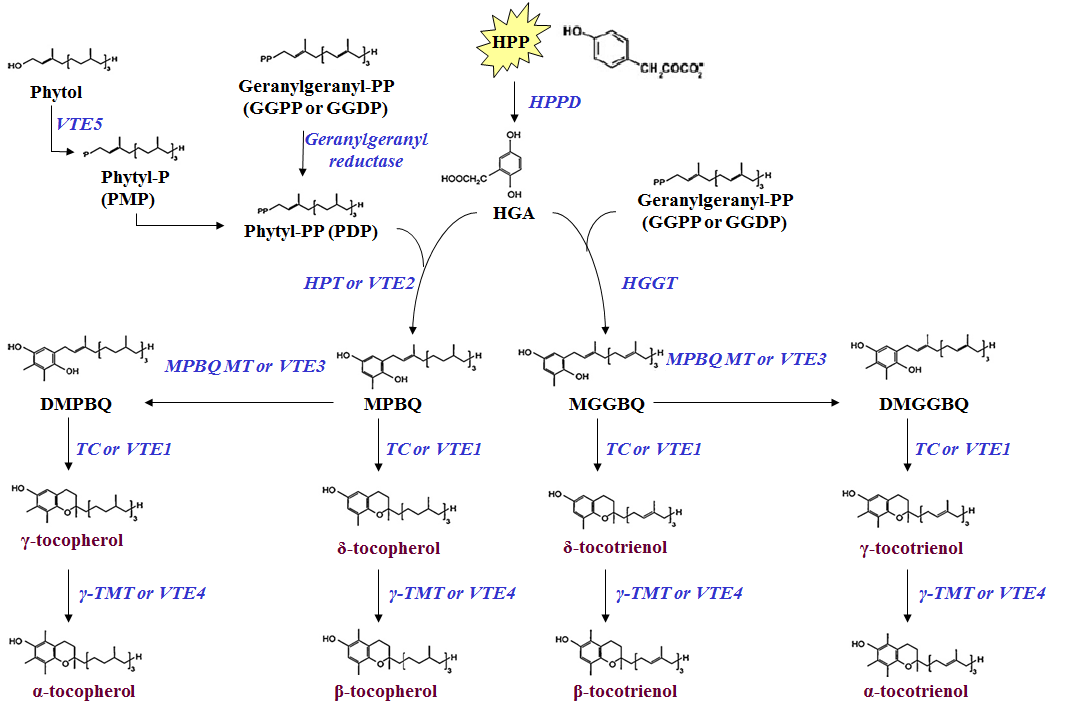
*

**Supplementary Figure S1.** Tocochromanol biosynthetic pathway in olive fruit (Figure modified from Georgiadou et al., 2015).

**
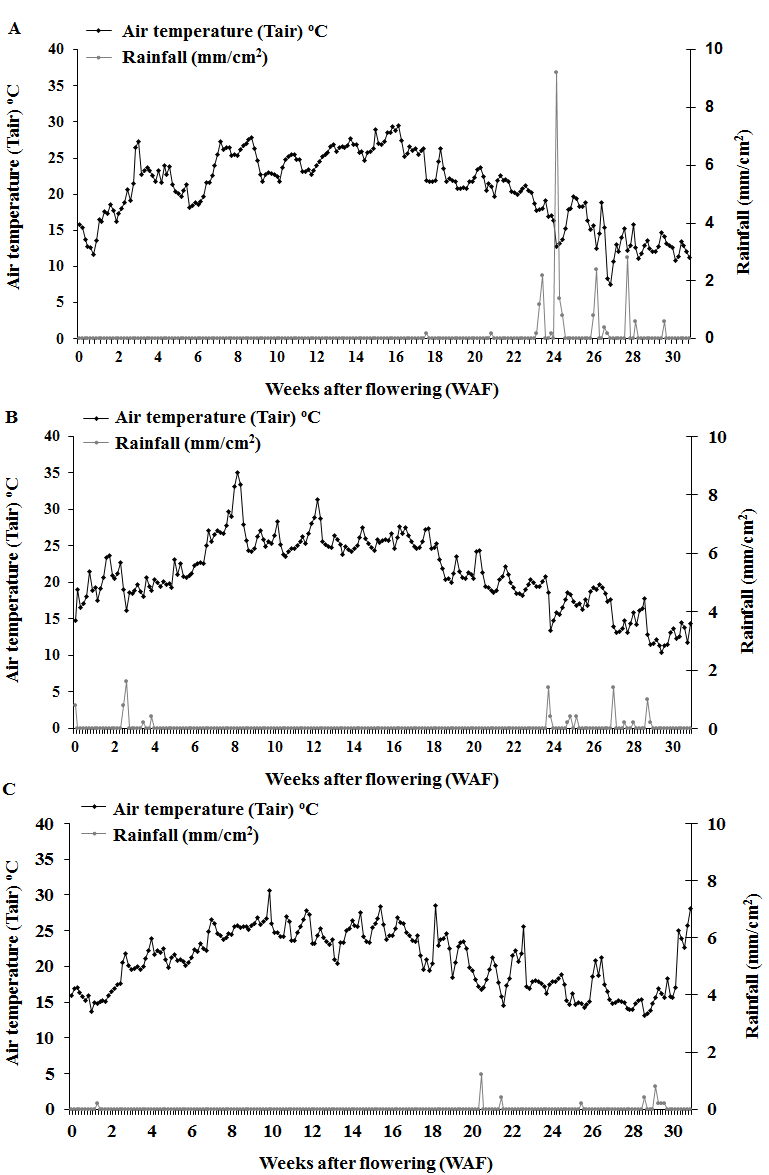
**

**Supplementary Figure S2.** Air temperature (Tair) °C and rainfall during the 30 weeks’ period after flowering for each of the three successive years (A, 1^st^ year; B, 2^nd^ year; C, 3^rd^ year) in the experimental orchard. Full bloom was at the end of May for every successive year and corresponds to 0 WAF.


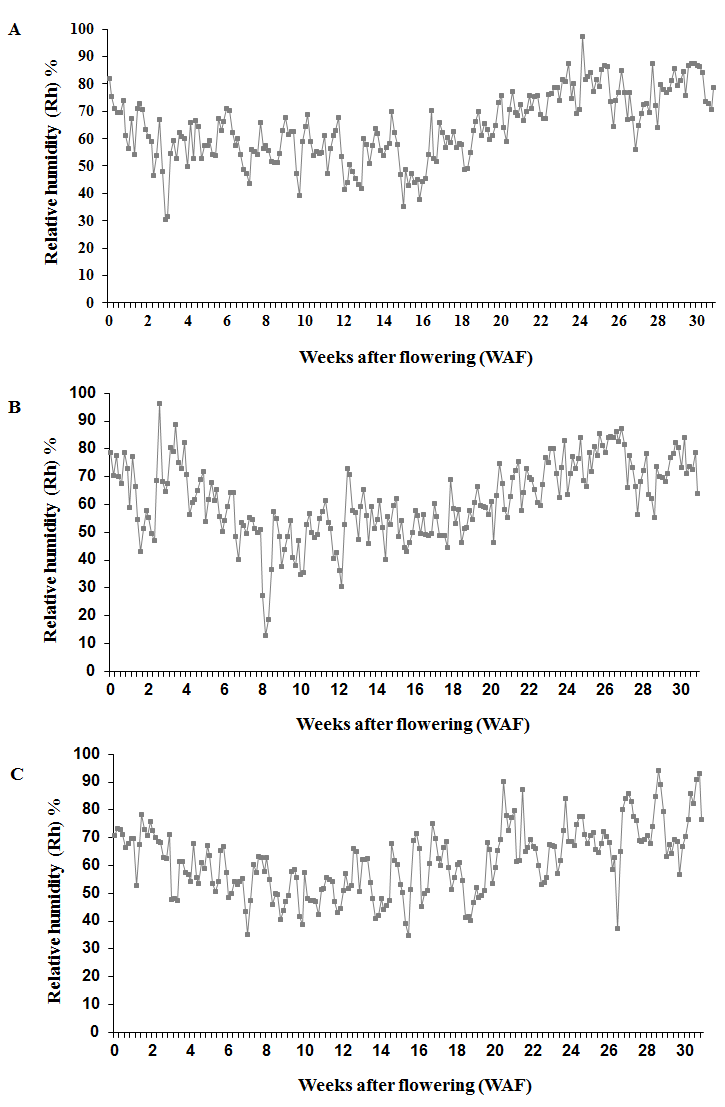


**Supplementary Figure S3.** Air relative humidity (Rh) % during the 30 weeks’ period after flowering for each of the three successive years (A, 1^st^ year; B, 2^nd^ year; C, 3^rd^ year) in the experimental orchard. Full bloom was at the end of May for every successive year and corresponds to 0 WAF.

**0.1**

gi 672143993 ref XP 008795886.1 ***Phoenix dactylifera***

gi 672143997 ref XP 008795888.1 ***Phoenix dactylifera***

gi 573941213 ref XP 006653001.1 ***Oryza brachyantha***

gi 357166664 ref XP 003580789.1 ***Brachypodium distachyon***

gi 76443933 gb ABA42674.1 ***Triticum aestivum***

**0.887**

**0.091**

gi 514742839 ref XP 004960241.1 ***Setaria italica***

gi 414584872 tpg DAA35443.1 ***Zea mays***

gi 76443929 gb ABA42672.1 ***Zea mays***

**0.779**

**0.850**

gi 694996876 ref XP 009396497.1 ***Musa acuminata* subsp. *malaccensi***

gi 694996874 ref XP 009396489.1 ***Musa acuminata* subsp. *malaccensi***

**0.921**

**0.797**

gi 702423130 ref XP 010067215.1 ***Eucalyptus grandis***

gi 223527316 gb EEF29465.1 ***Ricinus communis***

gi 743832498 ref XP 011024295.1 ***Populus euphratica***

gi 550332291 gb ERP57294.1 ***Populus trichocarpa***

**0.824**

gi 460378306 ref XP 004234914.1 ***Solanum lycopersicum***

gi 565397736 ref XP 006364441.1 ***Solanum tuberosum***

gi 698466854 ref XP 009783010.1 ***Nicotiana sylvestris***

gi 697166062 ref XP 009591844.1 ***Nicotiana tomentosiformis***

**0.907**

**0.746**

**0.917**

gi 508727027 gb EOY18924.1 ***Theobroma cacao***

gi 508727028 gb EOY18925.1 ***Theobroma cacao***

**0.993**

**0.816**

gi 225448861 ref XP 002269950.1 ***Vitis vinifera***

gi 731370145 ref XP 010665628.1 ***Beta vulgaris* subsp. *vulgaris***

**0.745**

gi 643739241 gb KDP45055.1 ***Jatropha curcas***

gi 641836336 gb KDO55302.1 ***Citrus sinensis***

**0.746**

***VTE5*** ***Olea europaea***

gi 645224089 ref XP 008218941.1 ***Prunus mume***

gi 657967897 ref XP 008375637.1 ***Malus domestica***

gi 470142948 ref XP 004307151.1 ***Fragaria vesca* subsp. *vesca***

gi 229315933 gb ACP43458.1 ***Lactuca sativa***

**0.827**

**0.946**

**0.604**

**0.704**

**0.748**

gi 659113424 ref XP 008456566.1 ***Cucumis melo***

gi 449493261 ref XP 004159238.1 ***Cucumis sativus***

**0.242**

gi 747093360 ref XP 011094482.1 ***Sesamum indicum***

gi 747093358 ref XP 011094481.1 ***Sesamum indicum***

**0.947**

**0.862**

**0.757**

**0.637**

**0.913**

gi 657404741 gb KEH43364.1 ***Medicago truncatula***

gi 561017491 gb ESW16295.1 ***Phaseolus vulgaris***

gi 502118218 ref XP 004496158.1 ***Cicer arietinum***

gi 571480813 ref XP 006588440.1 ***Glycine max***

gi 734312756 gb KHN00934.1 ***Glycine soja***

**0.917**

gi 729395091 ref XP 010552940.1 ***Tarenaya hassleriana***

gi 674963988 emb CDX70220.1 ***Brassica napus***

gi 377657567 gb AFB74217.1 ***Brassica napus***

gi 727639359 ref XP 010490961.1 ***Camelina sativa***

gi 727495047 ref XP 010423571.1 ***Camelina sativa***

gi 332003369 gb AED90752.1 ***Arabidopsis thaliana***

gi 727560253 ref XP 010452347.1 ***Camelina sativa***

**0.492**

**0.829**

**0.818**

**0.417**

**0.893**

**0.989**

**0.677**

gi 502129409 ref XP 004500292.1 ***Cicer arietinum***

gi 502129411 ref XP 004500293.1 ***Cicer arietinum***

gi 720028046 ref XP 010264775.1 ***Nelumbo nucifera***

gi 734316131 gb KHN02090.1 ***Glycine soja***

gi 76443939 gb ABA42677.1 ***Glycine max***

**0.913**

**0.765**

**0.654**

**1.000**

**0.622**

**0.824**

**Poaceae**

**Solanaceae**

Monocots

**Brassicaceae**

**Rosaceae**

**Fabaceae**

***Phytol kinases 2,3***

Dicots

**Supplementary Figure S4.** Phylogenetic tree of VTE5 protein homologues from higher plants.

**
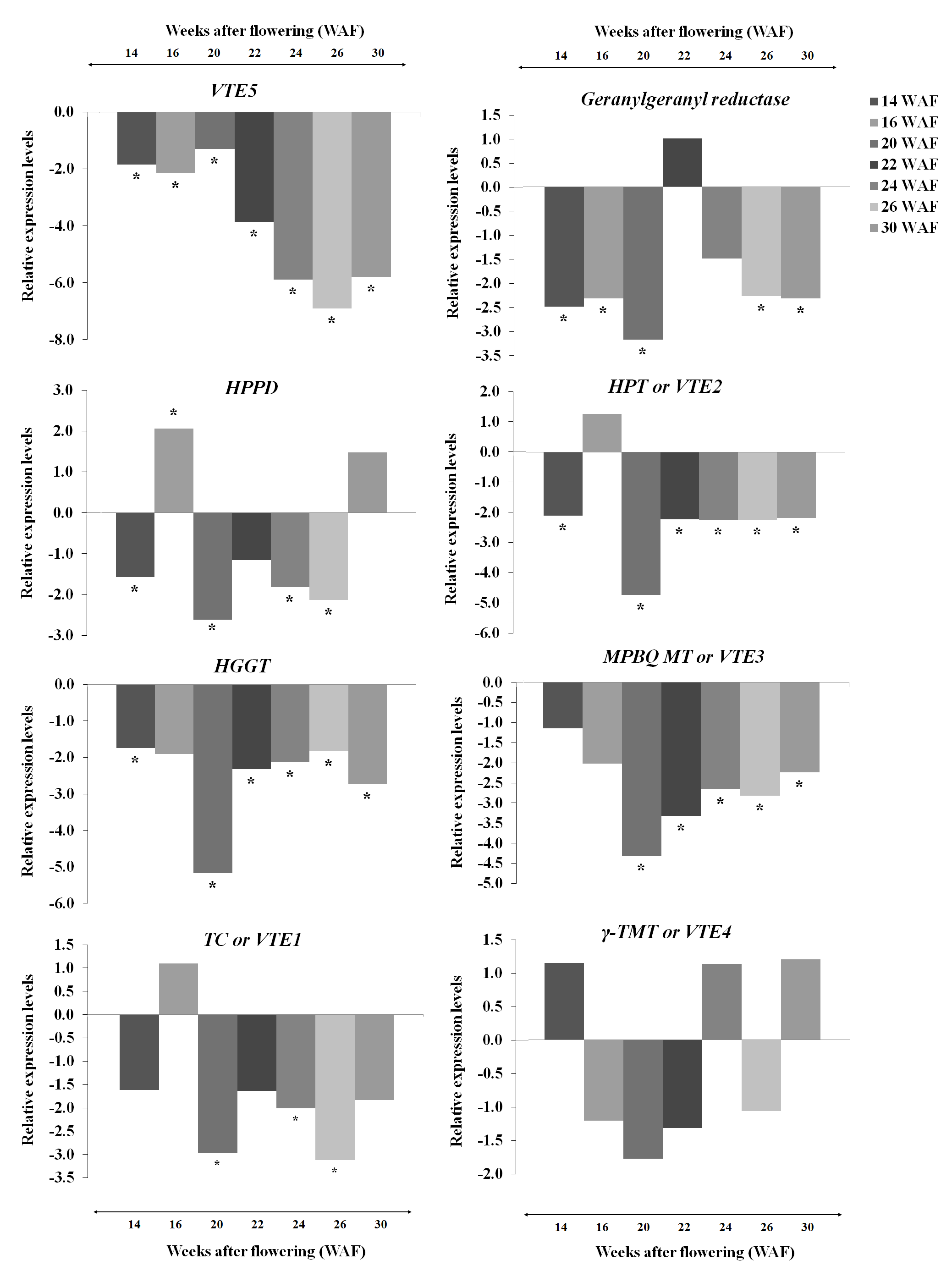
**

**Supplementary Figure S5.** Relative transcript levels of vitamin E biosynthesis genes (*VTE5, geranylgeranyl reductase, HPPD, VTE2, HGGT, VTE3, VTE1* and *VTE4*) in olive fruit (cv. ‘Koroneiki’) during 14-30 WAF (n = 3) during the 1^st^ year. Values that differ from the seasonal phase 10 WAF for the 1^st^ year, used as reference, with significance level P ≤ 0.05 are marked with *. Data are based on a statistical analysis of the means of three replications (Pfaffl et al., 2002).


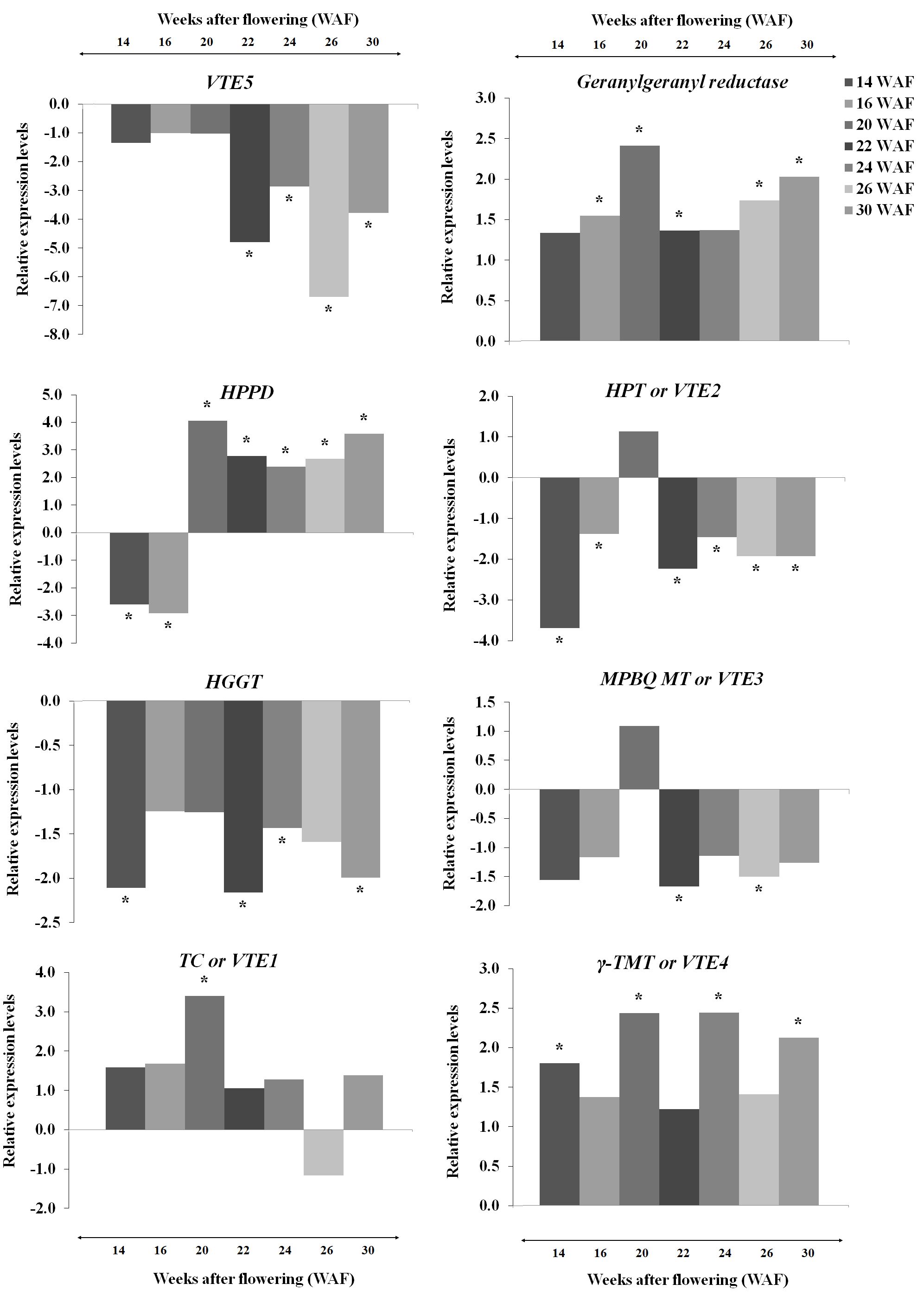


**Supplementary Figure S6.** Relative transcript levels of vitamin E biosynthesis genes (*VTE5, geranylgeranyl reductase, HPPD, VTE2, HGGT, VTE3, VTE1* and *VTE4*) in olive fruit (cv. ‘Koroneiki’) during 14-30 WAF (n = 3) during the 2^nd^ year. Values that differ from the seasonal phase 10 WAF for the 2^nd^ year, used as reference, with significance level P ≤ 0.05 are marked with *. Data are based on a statistical analysis of the means of three replications (Pfaffl et al., 2002).


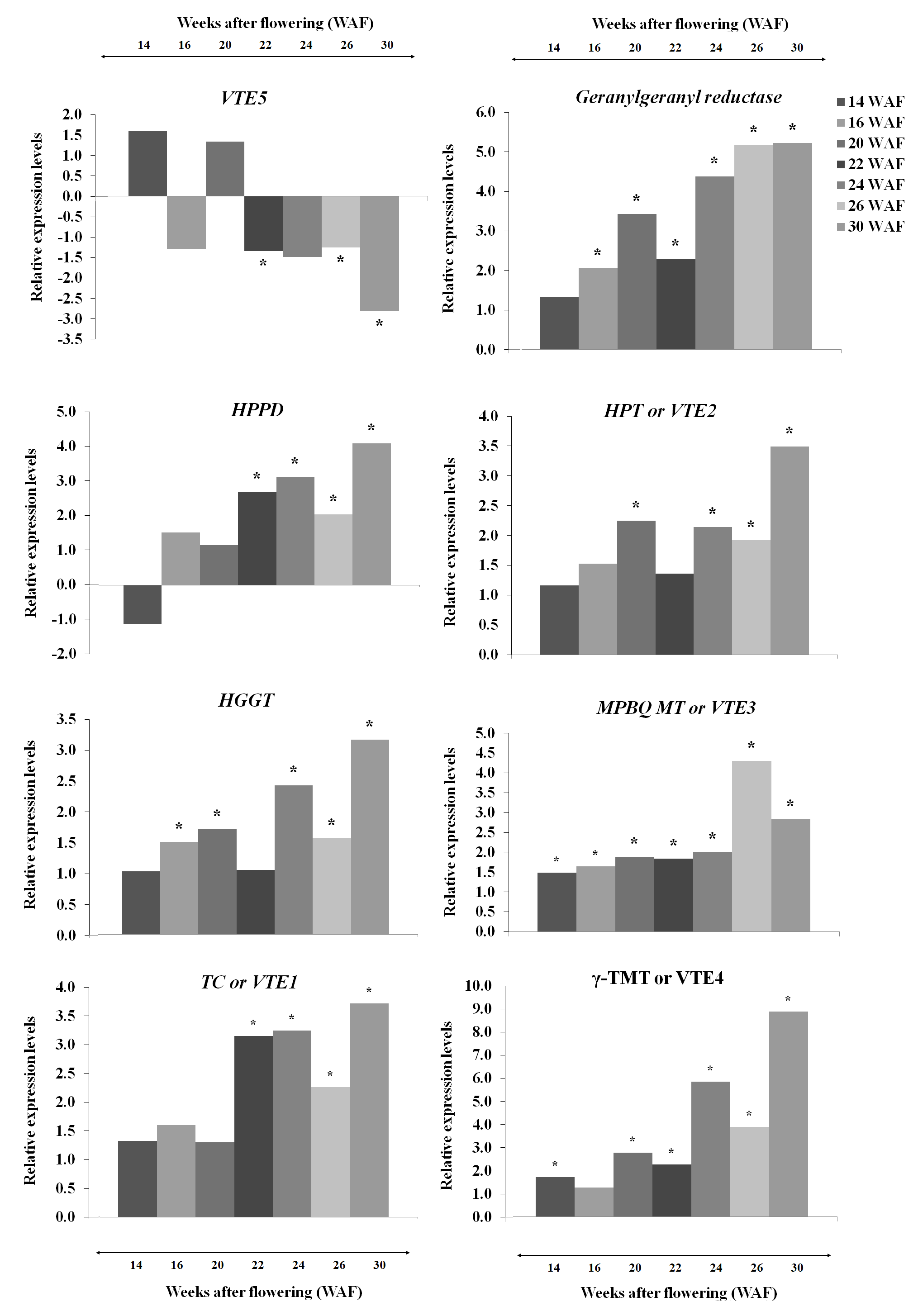


**Supplementary Figure S7.** Relative transcript levels of vitamin E biosynthesis genes (*VTE5, geranylgeranyl reductase, HPPD, VTE2, HGGT, VTE3, VTE1* and *VTE4*) in olive fruit (cv. ‘Koroneiki’) during 14-30 WAF (n = 3) during the 3^rd^ year. Values that differ from the seasonal phase 10 WAF for the 3^rd^ year, used as reference, with significance level P ≤ 0.05 are marked with *. Data are based on a statistical analysis of the means of three replications (Pfaffl et al., 2002).

**
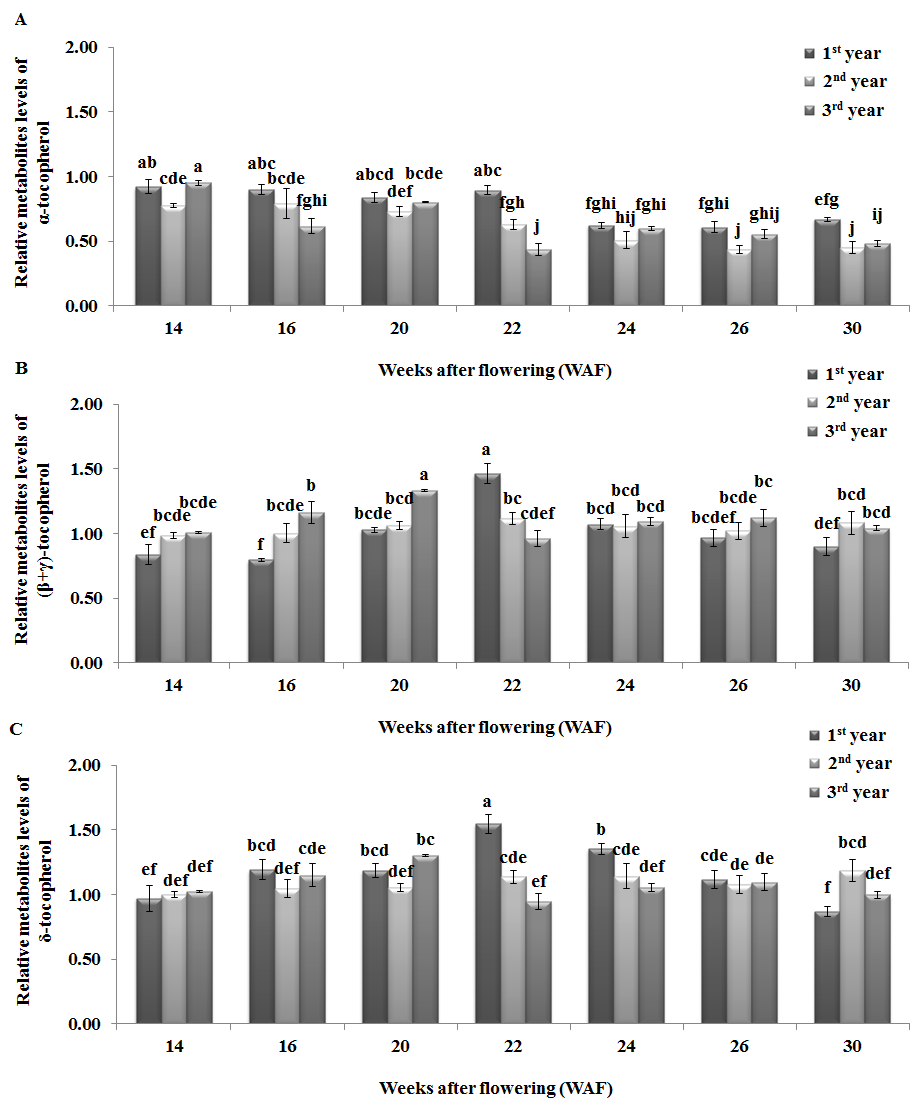
**

**Supplementary Figure S8.** Relative metabolite levels compared with 10 WAF for each year, of tocopherols in olive fruit (cv. “Koroneiki”) during 14-30 WAF (n = 3) during three successive years. Values followed by the same letter are not significantly different according to Duncan’s multiple range test at significance level 5% (P ≤ 0.05). Data are the means of three replications ± SE.


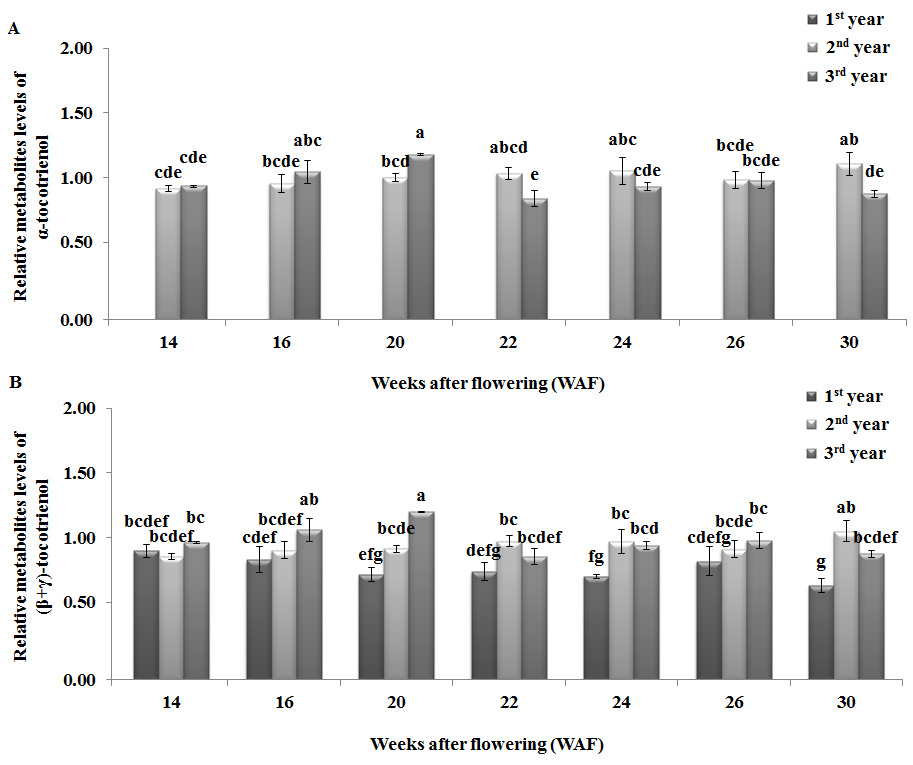


**Supplementary Figure S9.** Relative metabolite levels compared with 10 WAF for each year, of α-tocotrienol and (β+γ)-tocotrienol in olive fruit (cv. “Koroneiki”) during 14-30 WAF (n = 3) during three successive years. Alpha-tocotrienol during the 1^st^ year and δ-tocotrienol were non-detectable. Values followed by the same letter are not significantly different according to Duncan’s multiple range test at significance level 5% (P ≤ 0.05). Data are the means of three replications ± SE.


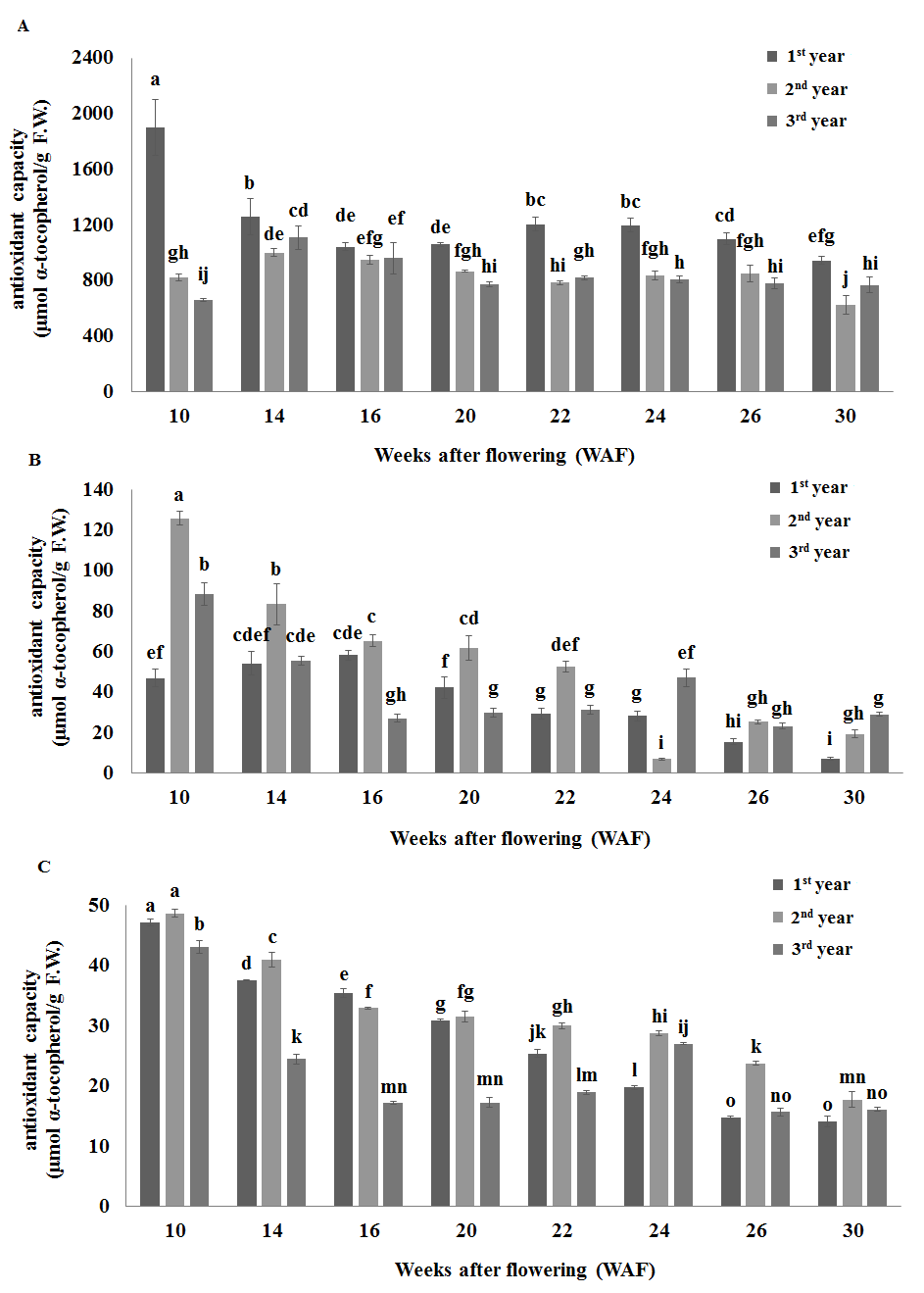


**Supplementary Figure S10.** Antioxidant capacity of olive fruits evaluated with A) the ferric reducing/antioxidant power (FRAP) method, B) 1,1-Diphenyl-2-picrylhydrazyl (DPPH) method and C) 2,2'-azino-bis(3-ethylbenzothiazoline-6-sulphonic acid) (ABTS) method (n = 3) during three successive years. Values followed by the same letter are not significantly different according to Duncan’s multiple range test (P ≤ 0.05). Data are the means of three replications ± SE.
